# Phylogenetic Lineages and Postglacial Dispersal Dynamics Characterize the Genetic Structure of the Tick, *Ixodes ricinus*, in Northwest Europe

**DOI:** 10.1371/journal.pone.0167450

**Published:** 2016-12-01

**Authors:** Knut H. Røed, Kjersti S. Kvie, Gunnar Hasle, Lucy Gilbert, Hans Petter Leinaas

**Affiliations:** 1 Department of Basic Sciences and Aquatic Medicine, Norwegian University of Life Sciences, Oslo, Norway; 2 Oslo Travel Clinic, St Olavs plass 3; Oslo, Norway; 3 James Hutton Institute, Macaulay Drive, Craigiebuckler, Aberdeen, United Kingdom; 4 Department of Biosciences, University of Oslo, Oslo, Norway; University of Minnesota, UNITED STATES

## Abstract

Dispersal and gene flow are important mechanisms affecting the dynamics of vectors and their pathogens. Here, patterns of genetic diversity were analyzed in many North European populations of the tick, *Ixodes ricinus*. Population sites were selected within and between areas separated by geographical barriers in order to evaluate the importance of tick transportation by birds in producing genetic connectivity across open sea and mountain ranges. The phylogenetic analyses of the mitochondrial control region and the cytochrome *b* gene revealed two distinct clades with supported sub-clades, with three genetic lineages: GB and WNo associated with Great Britain and western Norway respectively, and Eu with a wider distribution across continental Europe in agreement with much lower efficiency of tick dispersal by birds than by large mammals. The results suggest different ancestry of *I*. *ricinus* colonizing Britain and the rest of northern Europe, possibly from different glacial refuges, while ticks from western Norway and continental Europe share a more recent common ancestry. Demographic history modeling suggests a period of strong increase in tick abundance coincident with progression of the European Neolithic culture, long after their post-glacial colonization of NW Europe.

## Introduction

Ticks (Acari; Ixodidae) are bloodsucking ectoparasites of most terrestrial vertebrates, and they have a great impact on the public health and rural economy in many parts of the world [1]. *Ixodes ricinus* is a widespread and common European tick species that infests both birds and mammal hosts, and is an important vector for a wide range of pathogens. The tick-borne encephalitis virus (TBEV) complex and the *Borrelia* complex of spirochetes may cause serious diseases in humans [2, 3] while a series of other pathogens mainly represent problems for livestock [4, 5]. *I*. *ricinus* with associated pathogens are currently expanding in northern Europe, probably due to a combination of climate and habitat change and expansion of host species [6].

Dispersal and patterns of gene flow are crucial parameters for understanding the biology and infection pathways of pathogens in general [7]. In vector-borne pathogens, gene flow and thus the genetic diversity and structure are linked to the mobility and behavior of the vector, or to host transportation of vectors with low mobility such as ticks [8]. Hosts with the greatest potential for dispersing ticks include large mammals and birds. This may produce considerable genetic connectivity over broad spatial scales with little genetic structure or an isolation-by-distance pattern [9, 10]. However, previous range expansions and major topographic features can leave lasting genetic signatures on contemporary patterns of spatial genetic structure that persist, even at high rates of gene flow [11, 12].

Ixodid tick activity is inhibited by low temperatures, limiting their geographical distribution [13, 14]. Thus, the in the current Holocene epoch range of *I*. *ricinus* in central and northern Europe is the result of post-glacial range expansions from southern refuges. Currently *I*. *ricinus* has a continuous distribution across most of continental Europe [15]. Its northwestern range, however, is fragmented by seas and, at smaller scales, by fjords and mountainous regions: This may represent obstacles to tick dispersal and lead to a non-continuous distribution with isolated tick populations possibly being exposed to genetic drift and subsequent genetic sub-structuring. Such effects, however, will depend on the ability of the tick hosts to successfully cross open sea and mountain areas.

Phylogenies that span large portions of a species’ range offer a powerful means to elucidate important historical or contemporary processes that shape genetic diversity and structure [16]. Furthermore, the pattern of sequence differences in a gene genealogy contains information on the demographic history that can elucidate colonization events, effective dispersal or genetic barriers [17]. During the last few decades, genetic markers characterizing population structures have been used to improve estimates of present and past dispersal patterns of tick species [8, 10, 18–20]. In *I*. *ricinus*, studies have documented substantial genetic differences between European and North African populations, but very little phylogeographic structure at both wide and local scales across large parts of continental Europe [10, 11, 19]. Homogeneity of European *I*. *ricinus* has been explained by recent range expansion and gene flow due to passive dispersal of ticks by hosts within a continuous population distribution [10]. However, a recent study comparing the geographically widely separated *I*. *ricinus* populations of Great Britain and Latvia did show marked genetic differences [12].

The genetic structure of *I*. *ricinus* in Scandinavia and how it relates to continental Europe and Great Britain has not been investigated, although distinct phylogeographical patterns could be expected from the extensive geographical features (fjords and mountains) acting as barriers to tick distributional expansion since last glacial period. Transportation across open seas depends on birds during spring and autumn migration [21, 22], and inspection of recently arriving birds at bird observatories along the southern coast of Norway have shown large numbers of ticks being transported from the south [23]. However, because birds mainly host immature ticks (nymphs and larvae) rather than adults [21, 24], little is known about the effects of such transportation on tick gene flow. One indication of limited effect of tick transportation by birds across the sea is the maintenance of a disjunctive geographical distribution of different strains of the tick-borne TBEV complex within this area, with western TBEV in continental Europe and louping ill virus (LIV) in Great Britain [25]. We would expect that high gene flow between areas would have mixed the geographical distribution of the two pathogens.

Our sampling was designed to evaluate the importance of transportation by migratory birds relative to mammals in producing genetic connectivity between continental Europe and the more topographically isolated island of Great Britain and the Scandinavian Peninsula. We hypothesized the existence of a genetic structure (i) between British and continental European *I*. *ricinus* populations owing to reduced gene flow across open seas, and (ii) between western and south-eastern Norway owing to the south Norwegian Langfjella mountain range which separates these two areas and functions as a barrier against mammal dispersal. In addition, since the ticks of the two Norwegian areas are likely to be differentially influenced by various routes of bird migration, we also tested the hypothesis that ticks from western Norway would show more genetic resemblance to British ticks while those from southeast Norway would be more similar to ticks from south of the Skagerrak. Lastly, we also estimated the timescale of postglacial re-colonization and population expansion of *I*. *ricinus*. The two first hypotheses were confirmed. However, although we detected clear sign of a gene flow between Great Britain and western Norway, the typical west Norwegian lineages were more closely related to the continental European lineage (grouped into a common clade B). The analyses implied a fairly sudden population expansion of W European *I*. *ricinus* long after the initial postglacial colonization of the species.

## Material and Methods

### Sample collection and DNA sequencing

**Fig 1:**
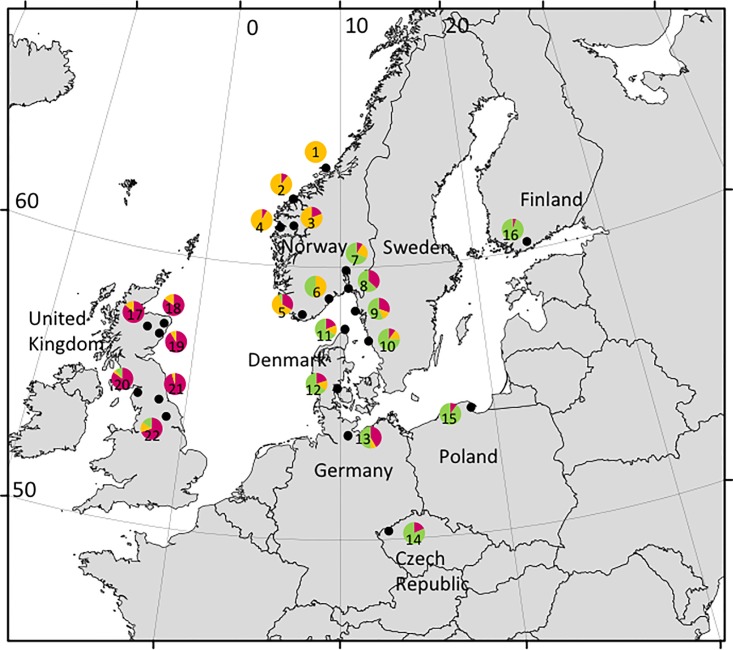
Sampling sites of *Ixodes ricinus*. Locations given as black dots. Pie diagrams show the proportion of the three mtDNA lineages for each site, with the GB lineage colored in burgundy, the WNo lineage in orange and the Eu lineage in green. Numbers within pie diagrams refer to the site number as given in Table 1. (Map made from www.naturalearthdata.com).

*Ixodes ricinus* were collected from 22 different sites in northern Europe (Fig 1 and S1 Table) with a sampling design focusing on the possible impact of geographical barriers such as open seas and mountain ranges in NW Europe. Most of the sites (128) were therefore placed in Britain and Scandinavia, while four additional sites were selected from Germany, Poland, the Czech Republic and Finland in order to relate the population genetics of *I*. *ricinus* from western European to more widespread patterns of continental Europe. The sites were sampled from 2001 to 2013 and the number of ticks from each site varied from 12 to 40. All ticks were collected from the vegetation by flagging technique, except samples from sites 1, 2 and 3 in western Norway, which had previously been sampled during 2001–2003 from hunted moose (*Alces alces*) or red deer (*Cervus elaphus*) in connection with a project led by the Norwegian Veterinary Institute. All ticks collected were immediately killed in 70% ethanol, where they were stored until analysis. Mostly adults, but also some nymphs, were sampled. Species identification was conducted according to [26] for adults and [27] for nymphs.

We used sequence differences in the mitochondrial DNA (mtDNA) to characterize the genetic structure of *I*. *ricinus*. MtDNA is a particularly suitable marker for analyzing genetic structure in ticks due to both maternal inheritance and because long distance dispersal is more common for adult females than adult males [9, 28]. DNA from individual ticks was extracted using DNeasy^TM^ Tissue Kit (Qiagen). Two fragments of mtDNA were amplified: parts of the cytochrome *b* (cyt *b*) gene and the control region (CR) using the primers as given by Casati *et al*. [19]. Polymerase Chain Reaction (PCR) was carried out in 25 μl volume using 0.12 μl AmpliTaq DNA polymerase (Applied Biosystems), 2.5 μl of the DNA extracts, 12.5 pmol of each primer, 200 μM of each dNTP, 2.5 μl 10 x PCR buffer (Applied Biosystems). The PCR protocol included an initial denaturation step at 95°C for 10 min, followed by 35 cycles with 95°C, 53°C and 72°C, each for 1 min, and a final 10 min extension step at 72°C. PCR products were purified with ExoSap-IT (Amersham), and sequenced using BigDye terminator chemistry version 1.1 on an ABI3100 automated sequencer (Applied Biosystems) following the manufacturer’s protocol. All sequences of the 336 bp cyt *b* gene and a 434 bp trimmed CR fragment have been deposited in GenBank (accession numbers KY025595-KY026024 and KY066742-KY067183; S2 Table). The CR fragment used in this study was a 427 bp fragment after deleting seven positions with gaps.

### Genealogical relationships among haplotypes

Genealogical reconstruction of haplotypes was analyzed for the amplified cyt b and CR fragments separately, and for the combined (concatenated) fragment. The best fitting model of sequence evolution of the concatenated fragment was determined using the Akaike information criterion as implemented in jModelTest [29]. The selected substitution model was HKY+I+G and this was used as a prior in Bayesian tree construction within the program BEAST v 1.7.5 [30] using the strict molecular clock with expansion growth as a coalescent prior in the model. Tests of tree likelihoods in TRACER 1.6 [31] gave preference for this model compared to constant size and exponential growth. Markov Chain Monte Carlo (MCMC) simulations were run with 5 x 10^7^ iterations, 10% burn-in and trees sampled every 5000 MCMC cycles. Log-files were inspected using TRACER and effective sample sizes (ESS) were used to evaluate MCMC convergence within chains. TreeAnnotater v.1.7.5 [30] was subsequently used to create a maximum clade credibility tree that represents the posterior distribution. Phylogenetic structure among haplotypes at the population level was analysed using a median-joining haplotype network implemented in Network v. 5.0 (www.fluxus-engineering.com).

### Substitution rates

Substitution rates for the mitochondrial CR and cyt *b* region do not currently exist for *I*. *ricinus*. Here, we estimated a population-level substitution rate for the concatenated fragments in *I*. *ricinus* based on the results from our phylogenetic analysis and paleoecological evidence. Most of the British Isles and Scandinavia was covered by ice during the last glacial maximum (LGM; 25–19 kybp (thousands of years before the present)) and re-colonization of potential tick host mammals occurred, at the earliest after the LGM in Britain and during the first part of the current Holocene epoch in Scandinavia [32, 33]. These inferences were consistent with the results of our phylogenetic analyses, with the tick populations in both Great Britain and western Norway being dominated by their respective haplotype groups, each with a star-like haplotype distribution pattern, suggesting common local ancestries. The mutation rate was assessed using BEAST with these haplotype groups separated into three taxon (one in Great Britain and two in western Norway), and the time of most recent common ancestor was used as the root calibration, in the tree construction. Due to the colder conditions during the Younger Dryas period (12.9–11.7 kybp), the calibration was modelled with a normal distribution with a mean (± SD) of 12 (± 4.5) kybp for the Great Britain haplotype group, while the mean prior distribution for the two western Norway haplotype groups were both set to 7 ± 2 kybp. To allow for rate variation among branches an uncorrelated lognormal relaxed-clock model was implemented. Tree prior, MCMC simulations, tree sampling and evaluation of MCMC convergence were used as in the haplotype genealogy analysis.

### Population genetic analysis

Genetic diversity was estimated as the number of haplotypes, haplotype diversity and nucleotide diversity calculated in DnaSP 5.10 [34]. ARLEQUIN v. 3.5 [35] was used to calculate pairwise F_ST_ differences. The program SAMOVA v. 1.0 [36] was used to define groups of populations that are spatially homogeneous and maximally genetically differentiated from each other. The program was run with a predefined number of groups (K) = 2–10 with 100 simulated annealing processes for 10 000 iterations.

### Historical demography

Signs of sudden demographic expansion (i.e. rapid population increase) were tested by using the mismatch distribution approach as implemented in ARLEQUIN with 10 000 bootstrap replicates. The sum of squared deviations was used to test the fit of the observed data to the model of sudden demographic expansion. We also performed two neutrality tests, Fu’s Fs and Fu and Li’s D [37, 38], implemented in DnaSP, to detect departure from mutation-drift equilibrium. The temporal trends in effective population size were reconstructed with Bayesian Skyline Plot (BSP) implemented in BEAST using the estimated clock rate and with MCMC simulations and tree sampling as described before.

### Data archiving

DNA sequences used in this study have been uploaded to GenBank (accession numbers KY025595-KY026024 for CR and KY066742-KY067183 for cyt *b*). Other datasets supporting this article have been uploaded as part of the Supporting Information.

## Results

### Genetic diversity

Mitochondrial cyt *b* and CR DNA were successfully amplified and sequenced from 442 and 430 *I*. *ricinus* specimens, respectively. Overall, the cyt *b* fragment showed nucleotide diversity ± SD of 0.0094 ± 0.0021 and haplotype diversity of 0.698 ± 0.0021 and defined 38 haplotypes. The CR showed nucleotide diversity of 0.0086 ± 0.0026 and haplotype diversity of 0.880 ± 0.009 defined by 81 haplotypes. The diversity for each sampling site for each fragment is given in S1 Table. Successful amplification of both fragments in the same individuals was obtained in 375 specimens, and the 763 bp concatenated fragments showed nucleotide and haplotype diversity of 0.009 ± 0.002 and 0.914 ± 0.008 and defined 101 haplotypes.

### Genealogical relationships among haplotypes

**Fig 2:**
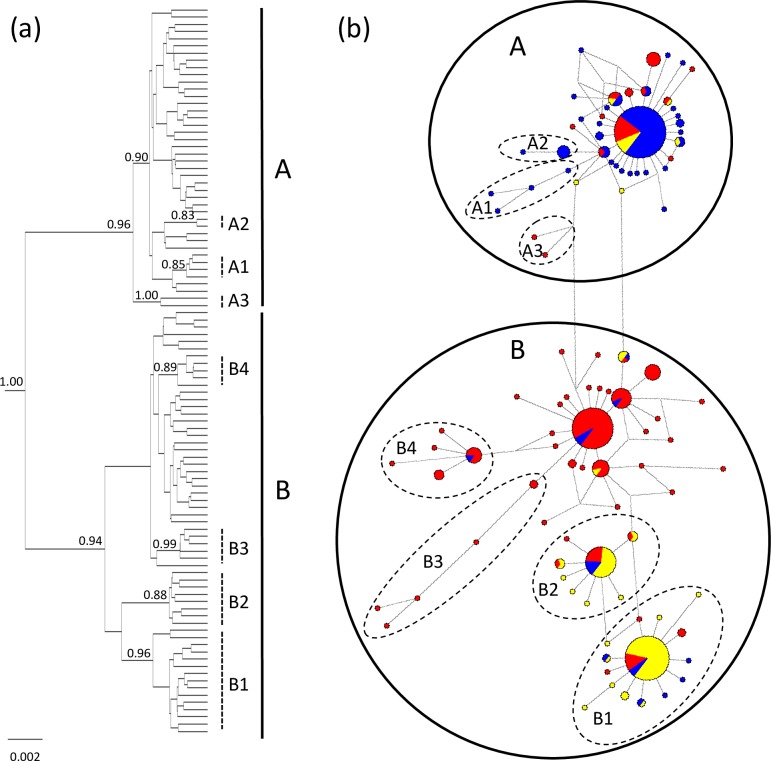
**Bayesian consensus tree (a) and network (b) for European *Ixodes ricinus*.** The estimates are based on 763 bp of concatenated cyt *b* and the control region of mtDNA. Bayesian posterior probabilities > 0.80 provided at the tree-nodes. Clade labels used throughout the text are indicated as vertical bars beside the tree and as encircled haplotypes in the network. The proportional occurrence of each haplotype in the material is illustrated by the size of the pie diagrams, which have colored pies according to the geographical distribution of the haplotypes among the three main areas of the study: blue = Great Britain, yellow = western Norway and red = continental Europe. (Not the difference in the meaning of the color codes in Figs 1 and 2).

The Bayesian phylogenetic tree (Fig 2A) reconstruction of the concatenated fragments particularly identified two relatively large monophyletic clades, denoted A and B, with posterior probabilities (pp) of 0.97 and 0.95 respectively, and both consisted of some highly supported sub-clades. The minimum spanning tree (Fig 2B) illustrates that clade A consisted of 42 haplotypes in 141 individuals, most of which (74%) were sampled at the six locations in Great Britain (from Scotland and England). This clade consisted of one haplotype at high frequency (0.53), with all other haplotypes radiating from this by one to four base pairs (Fig 2B). Such star-like patterns suggest that this haplotype to be ancestral [39]. The ancestral haplotype was dominant in all six British locations (frequency range 0.33–0.58). Two sub-clades (A1 and A2) nested within clade A, both with three mutations separated from the ancestral haplotype, were detected within Great Britain only (Fig 2B), while a third sub-clade (A3) separated from the ancestral haplotype with six mutations, consisted of two haplotypes found only outside Great Britain (Fig 2B). This possibly reflects a different ancestry of this sub-clade. Clade A, with the probable exception of sub-clade A3, is proposed to represent a distinct Great Britain (GB) lineage.

Clade B also contained haplotypes with distinct geographic structuring. The two highly supported sub-clades, B1 and B2 (Fig 2A) with15 and eight haplotypes respectively, both have a star-like haplotype pattern dominated by samples obtained from the five sites located along the west coast of Norway (i.e. 75% of the ticks sampled from site 1–5). This implies that sub-clades B1 and B2 represent a distinct west Norwegian (WNo) lineage. The remaining part of clade B (36 haplotypes in 122 individuals) cluster together in a network and are dominant in all samples except those from Great Britain and western Norway (Fig 2B). This cluster comprised several star-like subgroups (incl. subclades B_2_ and B_3_) with ancestral haplotypes possibly reflecting several sub-lineages. However, the genetic divergence of the polytomies was relatively shallow, which suggests that this cluster represents a distinct European mainland (Eu) lineage. Only 6% of the British samples and 4% of the western Norwegian samples clustered within the Eu lineage.

Separate reconstructions of the phylogenetic and minimum spanning trees for the 81 CR haplotypes gave similarly structured phylogenies as for the concatenated fragments, with pp of 0.99 for a clade dominated by GB haplotypes and pp of 1.00 and 0.87 for the two western Norwegian clades and 0.83 for a European mainland clade (S1 Fig). The phylogeny of the CR fragment identified B1 and B2 to represent separate clades, although the support for these to cluster with either clade A or B was low. The analyses of the 38 cyt *b* haplotypes, on the other hand, identified only two highly supported clades (both with pp of 0.99) dominated by haplotypes obtained in Great Britain and the rest of northwest Europe respectively (S2 Fig).

### Population genetic structure

The genetic differentiation among populations was strong with a structure largely following the geographical distribution of the three mtDNA lineages. This was confirmed by population pairwise F_ST_ differences for the concatenated fragment (Table 1). The genetic structure consisted of all six British sites (17–22) in one group, the five western Norwegian sites (1–5) in a second group and the continental Europe (site 6–16) including the eastern Norway, Sweden and Finland in a third group. The SAMOVA analysis gave similar results, with these three groups of populations (K = 3) best explained by the among-group spatial partitioning of molecular variance of the concatenated fragment (S3 Fig).

**Table 1:** Pairwise genetic differences in concatenated fragment of mtDNA CR and cyto *b* between samples of *Ixodes ricinus* from different locations in northwest Europe.

Site nr	1	2	3	4	5	6	7	8	9	10	11	12	13	14	15	16	17	18	19	20	21	22
01		*	**	***	***	***	***	***	***	***	***	***	***	***	***	***	***	***	***	***	***	***
02	.234		ns	ns	ns	***	***	***	***	***	***	***	***	***	***	***	***	***	***	***	***	***
03	.127	.000		ns	ns	**	***	***	**	***	***	***	***	***	*	***	***	***	***	***	***	***
04	.275	.000	.052		***	***	***	***	***	***	***	***	***	***	***	***	***	***	***	***	***	***
05	.178	.055	.000	.160		*	*	***	ns	***	*	ns	ns	**	*	***	***	***	***	***	***	ns
06	.242	.189	.127	.241	.107		ns	*	ns	ns	ns	ns	*	ns	ns	**	***	***	***	***	***	***
07	.286	.277	.189	.347	.129	.000		ns	ns	ns	ns	ns	*	ns	ns	ns	***	***	***	***	***	***
08	.399	.346	.251	.433	.145	.129	.046		ns	ns	ns	ns	ns	ns	ns	*	***	***	***	***	***	*
09	.324	.268	.166	.367	.058	.092	.037	.000		ns	ns	ns	ns	ns	ns	**	***	***	***	***	***	ns
10	.350	.318	.228	.392	.162	.001	.000	.060	.058		ns	ns	*	ns	ns	ns	***	***	***	***	***	***
11	.401	.366	.266	.460	.171	.057	.001	.027	.030	.000		ns	ns	ns	ns	ns	***	***	***	***	***	***
12	.296	.268	.165	.358	.084	.014	.000	.002	.000	.000	.000		ns	ns	ns	ns	***	***	***	***	***	*
13	.435	.336	.216	.473	.069	.205	.156	.035	.000	.171	.095	.075		ns	ns	***	**	***	**	ns	***	ns
14	.448	.375	.261	.491	.150	.067	.005	.000	.000	.009	.000	.000	.059		ns	Ns	***	***	***	***	***	*
15	.536	.460	.298	.607	.165	.077	.000	.000	.000	.000	.000	.000	.051	.000		Ns	***	***	***	***	***	*
16	.569	.522	.396	.604	.332	.112	.019	.105	.130	.011	.058	.053	.304	.049	.053		***	***	***	***	***	***
17	.741	.657	.535	.753	.403	.633	.576	.428	.351	.606	.550	.514	.212	.513	.604	.726		ns	ns	ns	ns	ns
18	.734	.658	.545	.747	.418	.640	.585	.440	.368	.613	.558	.524	.226	.526	.602	.719	.000		ns	ns	ns	*
19	.665	.584	.473	.681	.341	.571	.522	.382	.302	.549	.491	.455	.154	.456	.514	.651	.000	.000		ns	ns	ns
20	.641	.545	.426	.611	.279	.504	.453	.296	.220	.479	.404	.375	.070	.357	.415	.603	.005	.002	.000		ns	ns
21	.802	.729	.609	.813	.489	.708	.647	.504	.434	.678	.628	.560	.301	.616	.719	.795	.028	.004	.009	.047		***
22	.485	.373	.252	.506	.106	.324	.278	.145	.063	.305	.245	.193	.000	.185	.214	.434	.106	.120	.060	.006	.189	

Site numbers refers to locations in Fig 2. F_ST_ values are given below the diagonal and significant levels above. Significant levels given as ns = non-significant

*** = P <0.001

** = 0.001<P<0.01

* = 0.01< P <0.05.

Low or no differentiation was clearly the trend among samples within each of the three main areas. One exception to this was the island Hitra (site 1), which differed significantly from the other west Norwegian samples (Table 1). No genetic differences were detected among the Scottish samples collected during a period of 5 years. Similarly, there was little evidence for temporal effects among mainland western Norwegian samples obtained 8–10 years apart. Such genetic stability across generations suggests low genetic drift, at least within the present sampling periods, pointing towards rather large breeding populations. Population pairwise F_ST_ analyses for the CR fragment gave similar differentiation as for the concatenated fragment, while cyt *b* showed reduced differentiation between the west Norwegian locations and the continental European locations (S3 and S4 Tables). Thus, evolutionary time or selective pressure appears not to have been sufficient for the ticks to evolve genetic differentiation in this segment between the western Norway and its source European population.

The three population groups showed some degree of gene flow in the proportion of shared haplotypes, being 0.12 between Great Britain and western Norway, 0.10 between continental Europe and western Norway and 0.13 between Great Britain and continental Europe. When plotting the sampled populations according to their lineage composition on a map (Fig 1), both a south-north and east-west gradient became apparent. The Eu lineage appeared scant in the western Norwegian and the British material, with only 12.5% and 15% in two of the British sites. However, this lineage dominated at the rest of the Scandinavian sites where haplotypes of the WNo lineage were also common. Further to the east, in Czech Republic, Poland and Finland, the WNo lineage was not found. Worth noting, is also the significant presence of the GB lineage in all but two of our sites (Fig 1).

### Substitution rates and demographic history

The dating of the inferred evolutionary and demographic history of the three *I*. *ricinus* lineages is directly dependent on the estimated nucleotide substitution rates. Under the assumption that *I*. *ricinus* colonized Britain after the LGM and southwest Norway during the early Holocene at the earliest, we estimated a median substitution rate for the concatenated fragment of 0.283 s/s/My (substitution/site/million years) with 95% highest posterior density (HPD) intervals of 0.168–0.436 s/s/My. Separate analyses for the CR gave a median substitution rate of 0.479 s/s/My (HPD: 0.266–0.774 s/s/My) leaving an estimated rate of cyt *b* of 0.034 s/s/My. The analyses for the concatenated fragment yielded a genetic distance between the two main clades A and B of 0.006 (Fig 2A). Applying the estimated substitution rate of 28.3%/site/My, the evolutionary separation between these clades occurred about 16 kybp.

**Fig 3:**
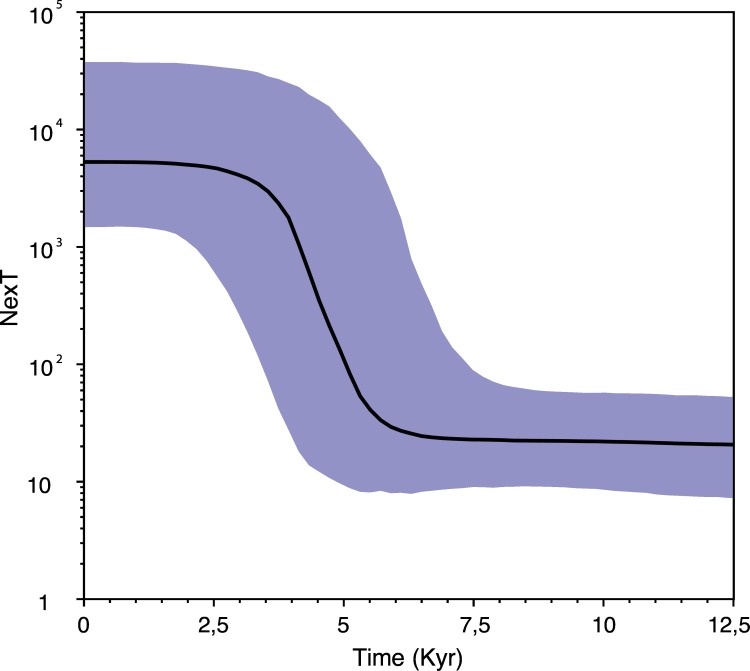
Bayesian skyline plot of temporal trends in effective population size of European ticks (*Ixodes ricinus*). The x-axis is given in thousands of years before present and the y-axis gives the product of effective population size and generation time (NexT). The black line represents the median estimate and the shaded area represents the 95% highest posterior density intervals.

The mismatched distribution analysis for the concatenated mtDNA fragment gave no significant deviation from the distribution expected from the sudden demographic expansion model for both the WNo and Eu lineages (Table 2). Highly significant Fu’s Fs and Fu and Li’s D (Table 2) support similar expansion in the GB lineage. Estimates of age of expansion revealed postglacial expansion for all lineages. The estimates could indicate a somewhat later expansion for the WNo lineage compared to the GB and Eu lineages (although there are overlapping 95% CI for the estimates; Table 2). Reconstruction of the demographic history with the Bayesian skyline plot on the combined dataset indicated rapid growth among the European tick population, which started approximately 6–5 kybp (Fig 3).

**Table 2:** Population genetic statistics for *Ixodes ricinus* mtDNA lineages.

					Nucleotide difference		Expansion time	
Lineage	Nh	SSD	Fu’s Fs	Fu and Li’s D	τ	CI (95%)	Years ago	CI (95%)
WNo	23	0.059^ns^	-28.93***	-2.54*	2.086	0.662–6.031	4830	1533–13965
Eu	36	0.0047^ns^	-34.43***	-1.94	3.350	2.154–6.602	7757	5988–15287
GB	40	0.045**	-73.81***	-3.45**	2.988	1.988–3.910	6919	4603–9054

Number of haplotypes (Nh), sum of square deviation (SSD), Fu’s Fs and Fu and Li’s D with corresponding P values (* p < 0.05, ** p < 0.02, *** p < 0.001), and estimate of nucleotide differences (τ) with 95% confidence interval (CI) are given. Estimates of age of sudden demographic expansion are based on the present estimate of mutation rate of 0.283 s/s/My.

## Discussion

Our spatial analyses showed low genetic differentiation in *I*. *ricinus* within large areas of continuous landmasses, but distinct divergent lineages between three main areas separated by geographical barriers against mammal migration; i.e. Great Britain, Western Norway and the rest of NW Europe. The genetically isolated position of the GB-lineage is quite striking in light of the short distance separating Great Britain from the European mainland. In Norway, the geographical separation of the two lineages WNo and Eu is consistent with the hypothesis that the south Norwegian mountain range is an efficient barrier against tick dispersal. However, as the WNo-lineage appears to have a continental European origin, our hypothesis about similarities between the WNo and GB tick lineages was not supported.

Spatial genetic structuring generally reflects a combination of historical vicariance, geographic isolation and ecological characteristics of the species [40, 41] Other studies have shown little genetic mtDNA structuring among *I*. *ricinus* populations of continental Europe at both local and regional scales, but substantial differences between North African and European populations [9, 10, 19]. This discrepancy with our results may well reflect differences in sampling design, since the previous studies did not include the northwestern-most areas separated by open seas, as we did in our study. A recent study, however, has shown considerable genetic differences between populations from Great Britain and Latvia [12]. In line with their results we found that, of all populations analyzed, the Finnish population, of about the same longitude as Latvia, contained the lowest element of the GB lineage. However, our results also imply that the distinct separation of the GB and Eu clades is associated with the sea separating Great Britain from the European continent.

The clear effect of open sea and mountain ranges on tick genetic structuring emphasizes the importance of mammals relative to birds in successful dispersal of *I*. *ricinus*. In particular large mammals such as red deer, moose, roe deer (*Capreolus capreolus)* and wild boar (*Sus scrofa*) have great potential of transporting *I*. *ricinus* owing to their generally high abundance across much of NW Europe, their great dispersal ability, and often high infestation by adult ticks [42, 43]. Transport of ticks by mammals would probably have been even larger in the past, when most of continental Europe was covered by extensive forests. Although our results suggest a primary role of mammals in the spatial dynamics of *I*. *ricinus* within landmasses, the haplotype sharing between the distributional ranges of the three genetic tick lineages also revealed effects of transportation by birds. In particular, both the British and western Norwegian tick populations appeared affected by a two-way gene flow across the North Sea, which can be mediated only by migrant birds. In contrast, the gene flow appears more asymmetrical between Great Britain and the rest of Europe, with a markedly stronger element of the GB-lineage in most European populations, than the Eu-lineage in Great Britain. This might suggest that transportation of ticks between these two areas is influenced primarily by eastward spring migration from Great Britain and less during the westward autumn migration. Nevertheless, this gene flow mediated by birds has not been sufficiently strong to erase the signal of a more basic spatial genetic structuring.

Our estimates of nucleotide substitution rates were much higher than the conventional mutation rate of 1.1–1.2% per million years per linage often applied to arthropod mitochondrial DNA, a calibration based on [44]. The high substitution rates we found could be explained by the use of the non-protein coding CR region known to accumulate mutations faster than other mtDNA regions [45], as compared to using protein-coding and ribosomal markers as in Brower’s calibration [44]. Also, it is now widely recognized that mutation rates in recently diverged haplotypes at or near the population level may be much higher than phylogenetic rates [46, 47]. Furthermore, our estimated high substitution rate is based on the assumption of rather early tick colonization after the last glaciation, which would give conservative estimates of the substitution rate.

During the glaciation period, temperate host species were mostly restricted to southern Europe and geographically isolated in several refuges, leading to genetic differentiation that is still traceable across the present distributions of many species. For example, east-west genetic divisions across Europe have typically been attributed to expansions from Italian/Balkan and Iberian refuges [48, 49]. The main split in *I*. *ricinus* haplotypes into clades A and B, with an estimated time of separation probably prior to the colonization of Great Britain and continental northwest Europe, suggests that postglacial expansion of *I*. *ricinus* into these areas came from different ancestral populations, and with clade B reaching coastal western Europe after the opening of the English Channel.

The heterogeneous network of the haplotypes of clade B (Fig 1A) tells a rather complex history of continental European and Scandinavian *I*. *ricinus*. The star-like substructures with several ancestral haplotypes suggest several repeated colonization events and isolation processes with subsequent demographic expansions. As the genetic divergence of the different sub-clades was relatively shallow, it likely reflects post-glacial colonization and isolation events. The concatenated CR/cyt *b* fragment suggests a common ancestor of the WNo and the Eu lineage, and thus that the founding haplotypes of western Norway came from continental Europe. This could have occurred via migrating birds or through transport of ticks by large mammals moving from the east to the west. The earliest traces of ungulates in western Norway occur after about nine kybp [32]. During the Holocene warm period (9–5 kybp) large parts of mountain ranges separating eastern and western Norway became forested, facilitating mammal migration and thus tick transportation into western Norway. The cooler and drier climate starting about 5 kybp reestablished a barrier against migration of mammalian tick hosts across the mountains [32], and thereby isolating the western Norway tick population. At the time they colonized western Norway, the founding haplotypes of the WNo lineage may have been more common in the whole Skagerrak-Kattegat area, while haplotypes typically representing the Eu lineage may have subsequently become more dominant by a later dispersal by large mammals from the south and east. If this is the case, then the considerable element of the WNo lineage in eastern Norway, Sweden, Denmark and North Germany might represent a remnant of a lineage once dominant in these areas, rather than due to dispersal from western Norway. Lack of an established Eu lineage in western Norway may simply reflect that these holotypes reached eastern Norway after colder climates prevented tick dispersal across the mountains.

The mismatched distribution analyses suggested a sudden demographic expansion for all three lineages, estimated to have occurred about 7–8 kybp for the GB and Eu lineages and 4–5 kybp for the WNo lineage (Table 2). These estimates fit well with the known progression of the Neolithic Revolution. This was a period characterized by a radical transformation of societies and landscape due to agricultural development, including domestication of livestock. Animal farming and crop growing became widespread in most regions in northern Europe, including Great Britain, from circa 7–5 kybp [50], and was firmly established in western Norway during the late Neolithic (4.5–4 kybp) [51]. These early agricultural communities probably improved conditions for *I*. *ricinus* through increased landscape heterogeneity, favouring a more complex fauna of natural tick hosts and introducing livestock as new host animals for *I*. *ricinus*.

The fact that the spatial structuring of three genetic lineages has been maintained despite thousands of years of tick transportation by migratory birds across the North Sea and the English Channel [21, 52] is quite striking. One reason might be that birds are mainly infested by immature ticks (larvae and nymphs) [21]. Since survival rate between succeeding developmental stages of *I*. *ricinus* is low [53], the overall reproductive success of ticks dispersed by birds is likely to be much lower than that of adult ticks dispersed by large mammals. However, in spite of this, ticks transported by birds may nevertheless be important for dispersal of tick-borne pathogens, in particular across the open sea. Transmission of pathogens requires only that the infected ticks have one blood-meal in the new area, thus transmitting the pathogen to the resident host. Migratory birds have been documented to transport *I*. *ricinus* carrying *Borrelia* and *Babesia* to the coast of southern Norway [23], but it is difficult to evaluate the impact of such transportation on already established resident pathogens [23]. However, the TBE virus is expanding to the north and has recently reached southeast Norway [54], which most likely has occurred via tick transportation by birds across the Skagerrak [21].

Interestingly, within our study areas established occurrence of the tick-borne louping ill virus (LIV) is primarily restricted to tick populations of Great Britain, in addition to a few records of the British subtype from the most southwestern part of Norway [55]; i.e. where the clade A is also fairly common. In addition to the British LIV subtype, there are also specific subtypes of LIV in Spain and Ireland [25], and it would be interesting to test if tick populations from Spain and Ireland also group into Clade A, which would suggest a link between this clade and LIV viruses in general. By comparison, the western TBE virus occurs in areas dominated by the Eu-lineage [56]. More research is needed to elucidate possible co-evolutionary links between *I*. *ricinus* lineages and different strains of the TBEV complex

## Conclusion

The splitting of *I*. *ricinus* from NW Europe into genetically distinct clades and lineages revealed geographically structured dispersal patterns of the species, still showing signatures of differences in post-glacial dispersal routs and colonization patterns. Main dispersal of the species appears to have occurred via movements of large mammals over land, while open seas and mountain ranges act as geographical barriers. Considerable over-sea transportation of ticks by birds for thousands of years has not been sufficient to mask the ancient colonization history of *I*. *ricinus* in Great Britain and western Norway. The distinct clade A of Great Britain may reflect a post-glacial expansion from a different refuge than the more continental clade B. However, analyses of *I*. *ricinus* populations from more southern parts of Europe are needed to evaluate this further. A sudden demographic expansion of *I*. *ricinus* appears to have occurred at the time of the Neolithic Revolution, suggesting human agricultural activity as a main driver on the dynamics and post-glacial history of the species.

The results emphasize the potential importance of combining population genetic analyses of *I*. *ricinus* with the spatial distribution of its pathogens. A linkage between clade A and the LIV subtypes of TBEV seems possible. The most widespread zoonotic pathogen of *I*. *ricinus*, however, is the genetically diverse *Borrelia burgdorferi* s. l. complex [57]. So far, the information of the geographic distribution of its different genospecies is too fragmented to link them to specific genotypes or lineages of the tick, but in the future this might be a useful approach to increase our understanding of the epidemiology of the pathogens.

## Supporting Information

S1 Fig Bayesian consensus tree (a) and network (b) for mtDNA control region **in** European Ixodes ricinus.
(TIF)

S2 Fig Bayesian consensus tree (a) and network (b) for mtDNA cyt *b* gene in European *Ixodes ricinus*.
(TIF)

S3 Fig Summary of the results of spatial analysis of molecular variance (SAMOVA) using the concatenated cytochrome *b* and control region mtDNA of *Ixodes ricinus* populations. (TIF)

S1 Table Sampling locations, year and levels of genetic variability in the control region (CR) and cytochrome *b* (cyt *b*) gene of mtDNA in *Ixodes ricinus*. (DOCX)

S2 Table Sampling locations, sequence name and GeneBank accession number for control region (CR) and cytochrome *b* (cyt *b)* gene of mtDNA in *Ixodus ricinus*. (DOCX)

S3 Table Pairwise genetic differences in control region of mtDNA between samples of *Ixodus ricinus* from different locations in northern Europe. (DOCX)

S4 Table Pairwise genetic differences in cytochrome b gene of mtDNA between samples of *Ixodus ricinus* from different locations in northern Europe. (DOCX)

## References

[1] Jongejan F, Uilenberg G. The global importance of ticks. Parasitol. 2004;129: S3–S14. 10.1017/s0031182004005967 15938502

[2] Chomel B. Lyme disease. Rev Sci Tech OIE. 2015;34: 569–576. 10.20506/rst.34.2.2380 26601457

[3] Valarcher JF, Hagglund S, Juremalm M, Blomqvist G, Renstrom L, Zohari S, et al 2015. Tick-borne encephalitis. Rev Sci Tech OIE. 2015;34: 453–466. 10.20506/rst.34.2.2371 26601448

[4] Hasle G, Bjune GA, Christensson D, Roed KH, Whist AC, Leinaas HP. Detection of *Babesia divergens* in southern Norway by using an immunofluorescence antibody test in cow sera. Act Vet Scand. 2010;52: 55. 10.1186/1751-0147-52-55 PMC2959048 20925923

[5] Stuen S, Granquist EG, Silaghi C. *Anaplasma phagocytophilum*-a widespread multi-host pathogen with highly adaptive strategies. Front Cell Infect Microbiol. 2013;3: 31 10.3389/fcimb.2013.00031 23885337 PMC3717505

[6] Gray JS, Dautel H, Estrada-Pena A, Kahl O, Lindgren E. Effects of climate change on ticks and tick-borne diseases in Europe. Interdiscip. Perspect. Infect Dis. 2009; 593232. 10.1155/2009/593232 PMC2648658 19277106

[7] Criscione CD, Poulin R, Blouin MS. Molecular ecology of parasites: elucidating ecological and microevolutionary processes. Mol Ecol. 2005;14: 2247–2257. 10.1111/j.1365-294X.2005.02587.x 15969711

[8] Araya-Anchetta A, Busch JD, Scoles GA. Thirty years of tick population genetics: A comprehensive review. Infect Genet Evol. 2015;29: 164–179. 10.1016/j.meegid.2014.11.008 25461844

[9] De Meeus T, Beati L, Delaye C, Aeschlimann A, Renaud F. Sex-biased genetic structure in the vector of Lyme disease, *Ixodes ricinus*. Evolution. 2002;56: 1802–1807. 12389725 10.1111/j.0014-3820.2002.tb00194.x

[10] Noureddine R, Chauvin A, Plantard O. Lack of genetic structure among Eurasian populations of the tick *Ixodes ricinus* contrasts with marked divergence from north-African populations. Int J Parasitol. 2011;41: 183–192. 10.1016/j.ijpara.2010.08.010 20946897

[11] Ishida S, Taylor DJ. Mature habitats associated with genetic divergence despite strong dispersal ability in an arthropod. BMC Evol Biol. 2007; 7. 10.1186/1471-2148-7-52 PMC1852300 17407568

[12] Dinnis RE, Seelig F, Bormane A, Donaghy M, Vollmer SA, Feil EJ, et al Multilocus sequence typing using mitochondrial genes (mtMLST) reveals geographic population structure of *Ixodes ricinus* ticks. Ticks Tick Borne Dis. 2014;5: 152–160. 10.1016/j.ttbdis.2013.10.001 24361120

[13] Gilbert L. Altitudinal patterns of tick and host abundance: a potential role for climate change in regulating tick-borne diseases? Oecologia. 2010;162: 217–225. 10.1007/s00442-009-1430-x 19685082

[14] Gilbert L, Aungier J, Tomkins JL. Climate of origin affects tick (*Ixodes ricinus*) host- seeking behavior in response to temperature: implications for resilience to climate change? Ecol Evol. 2014;4: 1186–1198. 10.1002/ece3.1014 24772293 PMC3997332

[15] Estrada-Pena A, Farkas R, Jaenson TGT, Koenen F, Madder M, Pascucci I, et al Association of environmental traits with the geographic ranges of ticks (Acari: Ixodidae) of medical and veterinary importance in the western Palearctic. A digital data set. Exp Appl Acarol. 2013;59: 351–366. 10.1007/s10493-012-9600-7 22843316 PMC3557372

[16] Manel S, Holderegger R. Ten years of landscape genetics. Trends Ecol Evol. 2013;28: 614–621. 10.1016/j.tree.2013.05.012 23769416

[17] Emerson BC, Paradis E, Thébaud C. Revealing the demographic histories of species using DNA sequences. Trends Ecol Evol. 2001;16: 707–716.

[18] McCoy KD, Boulinier T, Tirard C, Michalakis Y. Host-dependent genetic structure of parasite populations: differential dispersal of seabird tick host races. Evolution. 2003;57: 288–296. 12683525 10.1111/j.0014-3820.2003.tb00263.x

[19] Casati S, Bemasconib MV, Gern L, Piffaretti JC. Assessment of intraspecific mtDNA variability of European *Ixodes ricinus* sensu stricto (Acari: Ixodidae). Infect Genet Evol. 2008;8: 152–158. 10.1016/j.meegid.2007.11.007 18206426

[20] Dietrich M, Kempe F, Boulinier T, McCoy KD. Tracing the colonization and diversification of the worldwide seabird ectoparasite *Ixodes uriae*. Mol Ecol. 2014;23: 3292–3305. 10.1111/mec.12815 24888342

[21] Hasle G, Bjune G, Edvardsen E, Jakobsen C, Linnehol B, Roer JE, et al Transport of Ticks by Migratory Passerine Birds to Norway. J Parasitol. 2009;95: 1342–1351. 10.1645/GE-2146.1 19658452

[22] Sparagano O, George D, Giangaspero A, Spitalska E. Arthropods and associated arthropod-borne diseases transmitted by migrating birds. The case of ticks and tick-borne pathogens. Vet Parasitol. 2015;213: 61–66. 10.1016/j.vetpar.2015.08.028 26343302

[23] Hasle G, Bjune GA, Midthjell L, Roed KH, Leinaas HP. Transport of *Ixodes ricinus* infected with *Borrelia* species to Norway by northward-migrating passerine birds. Ticks Tick Borne Dis. 2011;2: 37–43. 10.1016/j.ttbdis.2010.10.004 21771535

[24] James MC, Furness RW, Bowman AS, Forbes KJ, Gilbert L. The importance of passerine birds as tick hosts and in the transmission of *Borrelia burgdorferi*, the agent of Lyme disease: a case study from Scotland. Ibis. 2011;153: 293–302.

[25] Grard G, Moureau G, Charrel RN, Lemasson J-J, Gonzalez J-P, Gallian P, et al Genetic characterization of tick-borne flaviviruses: New insights into evolution, pathogenetic determinants and taxonomy. Virology. 2007;361: 80–92. 10.1016/j.virol.2006.09.015 17169393

[26] Hillyard PD. Ticks of North-West Europe. In: Barnes RSK, Crothers JH, editors. Synopses of the British Fauna (New Series), 1996. No 52.

[27] Morel PC, Perez C. Morphologie des stases préimaginales des Ixodidae s. str. d’Europe occidentale. V. Les nymphes des *Ixodes* s. str. Acarologia. 1977;19: 579–586.

[28] Kempf F, McCoy KD, De Meeus T. Wahlund effects and sex-biased dispersal in *Ixodes ricinus*, the European vector of Lyme borreliosis: New tools for old data. Infect Genet Evol. 2010;10: 989–997. 10.1016/j.meegid.2010.06.003 20601167

[29] Posada D. jModelTest: Phylogenetic model averaging. Mol Biol Evol. 2008;25: 1253–1256. 10.1093/molbev/msn083 18397919

[30] Drummond AJ, Suchard MA, Xie D, Rambaut A. Bayesian Phylogenetics with BEAUti and the BEAST 1.7. Mol Biol Evol. 2012;29: 1969–1973. 10.1093/molbev/mss075 22367748 PMC3408070

[31] Rambaut ASM, Xie D, Drummond AJ. Tracer v1.6. 2014; Available: http://beastbioedacuk/Tracer.

[32] Rosvold J, Halley DJ, Hufthammer AK, Andersen R, Minagawa M. The rise and fall of wild boar in a northern environment: Evidence from stable isotopes and subfossil finds. Holocene. 2010;20: 1113–1121.

[33] Montgomery WI, Provan J, McCabe AM, Yalden DW. Origin of British and Irish mammals: disparate post-glacial colonisation and species introductions. Q Sci Rev. 2014;98: 144–165.

[34] Rozas J, Sanchez-DelBarrio JC, Messeguer X, Rozas R. DnaSP, DNA polymorphism analyses by the coalescent and other methods. Bioinformatics 2003;19: 2496–2497. 14668244 10.1093/bioinformatics/btg359

[35] Excoffier L, Lischer HEL. Arlequin suite ver 3.5: a new series of programs to perform population genetics analyses under Linux and Windows. Mol Ecol Resour. 2010;10: 564–567. 10.1111/j.1755-0998.2010.02847.x 21565059

[36] Dupanloup I, Schneider S, Excoffier L. A simulated annealing approach to define the genetic structure of populations. Mol Ecol. 2002;11: 2571–2581. 12453240 10.1046/j.1365-294x.2002.01650.x

[37] Fu YX. Statistical tests of neutrality of mutations against population growth, hitchhiking and background selection. Genetics. 1997;147: 915–925. 9335623 10.1093/genetics/147.2.915 PMC1208208

[38] Fu YX, Li WH. Statistical tests of neutrality of mutations. Genetics. 1993;133: 693–709. 8454210 10.1093/genetics/133.3.693 PMC1205353

[39] Posada D, Crandall KA. MODELTEST: testing the model of DNA substitution. Bioinformatics. 1998;14: 817–818. 9918953 10.1093/bioinformatics/14.9.817

[40] Templeton AR. Nested clade anlyses of phylogeographic data: testing hypotheses about gene flow and population history Mol Ecol. 1998;7: 381–397. 9627999 10.1046/j.1365-294x.1998.00308.x

[41] Avise JC. Phylogeography: The History and Formation of Species. Cambridge: Harvard University Press; 2000.

[42] Handeland K, Qviller L, Vikoren T, Viljugrein H, Lillehaug A, Davidson RK. *Ixodes ricinus* infestation in free-ranging cervids in Norway -A study based upon ear examinations of hunted animals. Vet Parasitol. 2013;195: 142–149. 10.1016/j.vetpar.2013.02.012 23541678

[43] Kriz B, Daniel M, Benes C, Maly M. The Role of Game (Wild Boar and Roe Deer) in the Spread of Tick-Borne Encephalitis in the Czech Republic. Vector Borne Zoonotic Dis. 2014;14: 801–807. 10.1089/vbz.2013.1569 25409271 PMC4238255

[44] Brower AVZ. Rapid morphological radiation and convergence among races of the butterfliy *Heliconius arato* inferred from patterns of mitochondrial DNA evolution. Proc Natl Acad Sci USA. 1994;91: 6491–6495. 8022810 10.1073/pnas.91.14.6491 PMC44228

[45] Pesole G, Gissi C, Chirico AD, Saccone C. Nucleotide substitution rate of mammalian mitochondrial genomes. J Mol Evol. 1999;48: 427–434. 10079281 10.1007/pl00006487

[46] Ho SYW, Phillips MJ, Cooper A, Drummond AJ. Time dependency of molecular rate estimates and systematic overestimation of recent divergence times. Mol Biol Evol. 2005;22: 1561–1568. 10.1093/molbev/msi145 15814826

[47] Papadopoulou A, Anastasiou I, Vogler AP. Revisiting the Insect Mitochondrial Molecular Clock: The Mid-Aegean Trench Calibration. Mol Biol Evol. 2010;27: 1659–1672. 10.1093/molbev/msq051 20167609

[48] Hewitt GM. Some genetic consequences of ice ages, and their role in divergence and speciation. Biol J Linn Soc. 1996;58: 247–276.

[49] Taberlet P, Fumagalli L, Wust-Saucy AG, Cosson JF. Comparative phylogeography and postglacial colonization routes in Europe. Mol Ecol. 1998;7: 453–464. 9628000 10.1046/j.1365-294x.1998.00289.x

[50] Rowley-Conwy P. How the West was lost—A reconsideration of agricultural origins in Britain, Ireland, and southern Scandinavia. Curr Anthropol. 2004;45: S83–S113.

[51] Høgstøl M, Prøsch-Danielsen L. Impulses from agro-pastoralism in the 4th and 3rd millennia BC on the south-western coastal rim of Norway. Env Archaeol. 2006;11: 19–34.

[52] Pietzsch ME, Mitchell R, Jameson LJ, Morgan C, Medlock JM, Collins D, et al Preliminary evaluation of exotic tick species and exotic pathogens imported on migratory birds into the British Isles. Vet Parasitol. 2008;155: 328–332. 10.1016/j.vetpar.2008.05.006 18585865

[53] Randolph SE, Green RM, Hoodless AN, Peacey MF. An empirical quantitative framework for the seasonal population dynamics of the tick *Ixodes ricinus*. Int J Parasitol. 2002;32: 979–989. 12076627 10.1016/s0020-7519(02)00030-9

[54] Andreassen A, Jore S, Cuber P, Dudman S, Tengs T, Isaksen K, et al Prevalence of tick borne encephalitis virus in tick nymphs in relation to climatic factors on the southern coast of Norway. Parasit Vectors. 2012;5: 177 10.1186/1756-3305-5-177 22913287 PMC3497858

[55] Ytrehus B, Vainio K, Dudman SG, Gilray J, Willoughby K. Tick-Borne Encephalitis Virus and Louping-Ill Virus May Co-Circulate in Southern Norway. Vector Borne Zoonotic Dis. 2013;13: 762–768. 10.1089/vbz.2012.1023 23808981

[56] Barrett PN, Dorner F, Ehrlich HJ, Plotkin SA. Tick-borne encephalitis virus vaccine In: Plotkin SA, Orenstein WA, editors. Vaccines Elsevier Inc, USA, 2004 pp 773–788.

[57] Rauter C, Hartung T. Prevalence of *Borrelia burgdorferi* sensu lato genospecies in Ixodes ricinus ticks in Europe: a metaanalysis. Appl Env Microbiol. 2005;71: 7203–7216. 16269760 10.1128/AEM.71.11.7203-7216.2005 PMC1287732

