# Supplementary figures and images for: Phylogenetic Lineages and Postglacial Dispersal Dynamics Characterize the Genetic Structure of the Tick, Ixodes ricinus, in Northwest Europe
Source: PLoS One. 2016 Dec 1;11(12):e0167450. doi: 10.1371/journal.pone.0167450 (PMC5131986; doi:10.1371/journal.pone.0167450)

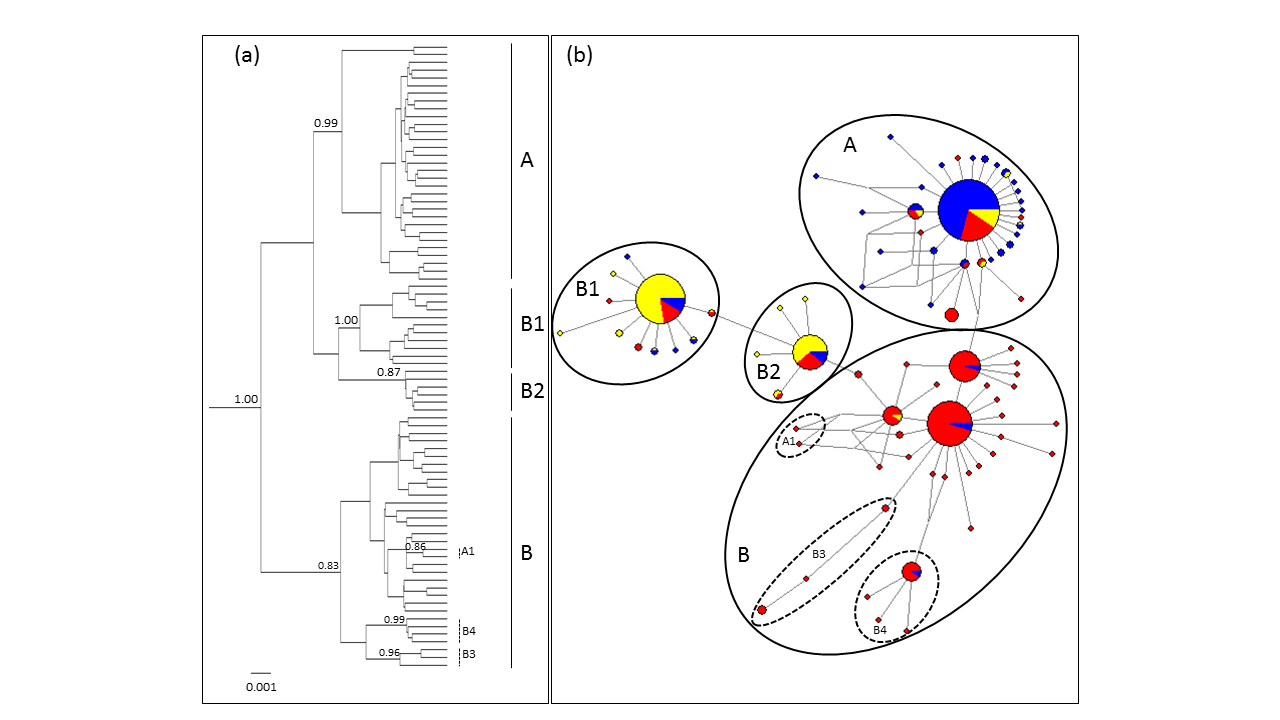

Supplement: S1 Fig — Bayesian consensus tree (a) and network (b) for mtDNA control region in European Ixodes ricinus. (TIF) [file pone.0167450.s001.tif]

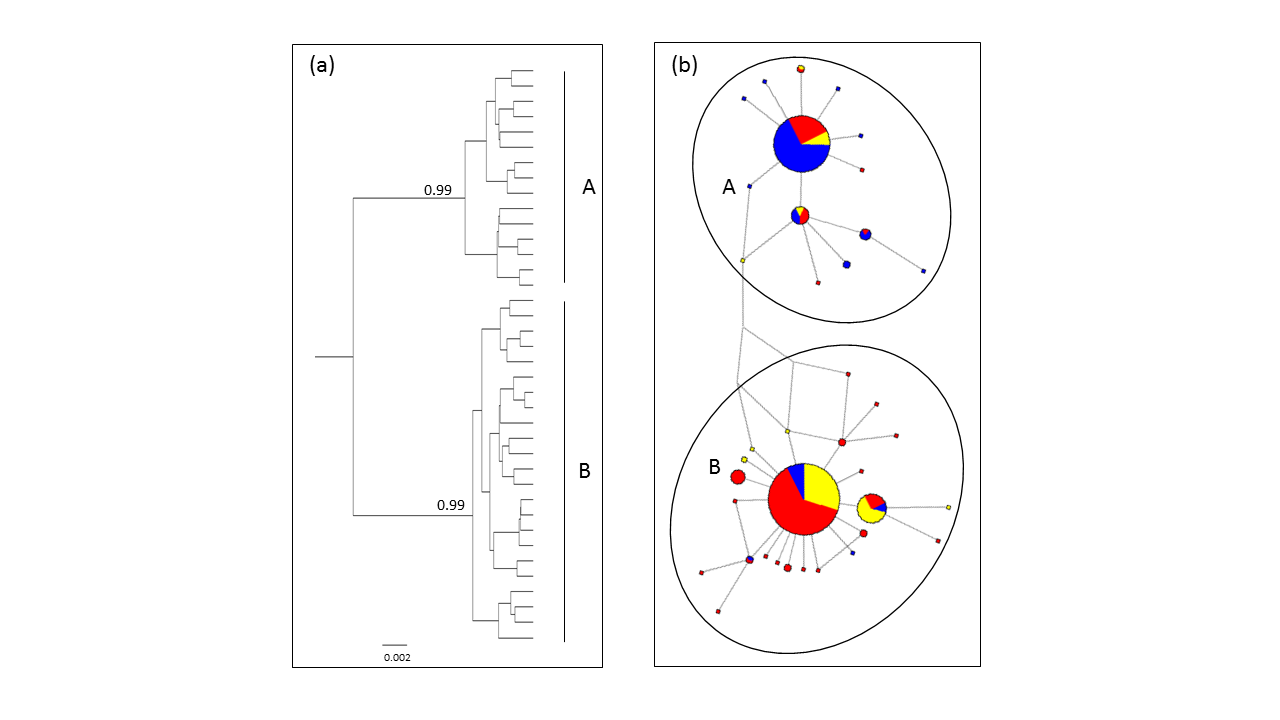

Supplement: S2 Fig — Bayesian consensus tree (a) and network (b) for mtDNA cyt b gene in European Ixodes ricinus. (TIF) [file pone.0167450.s002.tif]

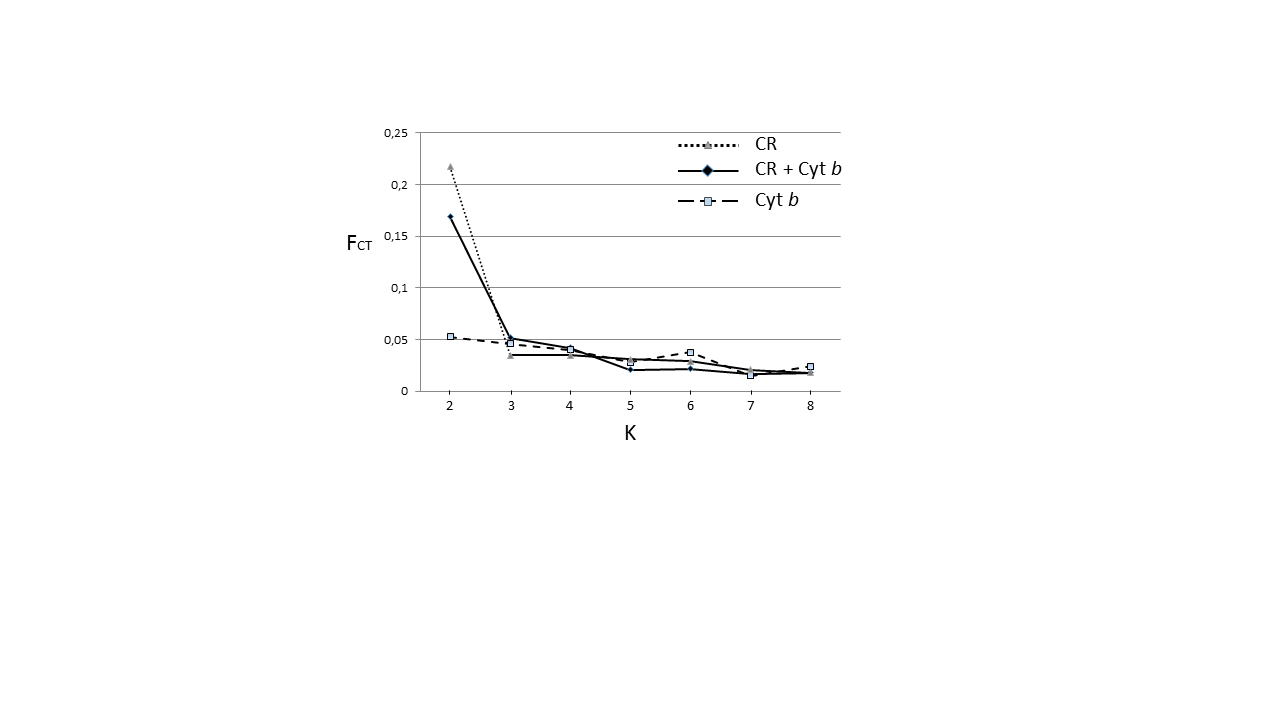

Supplement: S3 Fig — (TIF) [file pone.0167450.s003.tif]
