# Supplementary material for: Phylogenetic Lineages and Postglacial Dispersal Dynamics Characterize the Genetic Structure of the Tick, Ixodes ricinus, in Northwest Europe
Source: PLoS One. 2016 Dec 1;11(12):e0167450. doi: 10.1371/journal.pone.0167450 (PMC5131986; doi:10.1371/journal.pone.0167450)
Supplement: S1 Table — (DOCX) [file pone.0167450.s004.docx]

S1 Table. Sampling locations, year and levels of genetic variability in the control region (CR) and cytochrome *b* (cyt *b*) gene of mtDNA in *Ixodes ricinus*. N=number of individuals, nh = number of haplotypes. Nr. refers to the map location in Fig. 2.

|  |  |  |  | | CR | | | | | | cyt *b* | | | | |
| --- | --- | --- | --- | --- | --- | --- | --- | --- | --- | --- | --- | --- | --- | --- | --- |
| Nr. | Location | Country | Year of sampling | N | | nh | Gene  diversity | Nucleotide diversity | |  | | N | nh | Gene  diversity | Nucleotide  diversity |
| 1 | Hitra | Norway | 2001-2003 | 20 | | 5 | 0.663 | 0.0042 | |  | | 19 | 3 | 0.573 | 0.0017 |
| 2 | Hareide | Norway | 2001-2003 | 19 | | 4 | 0.520 | 0.0022 | |  | | 21 | 4 | 0.481 | 0.0058 |
| 3 | Gaular | Norway | 2001-2003 | 21 | | 7 | 0.771 | 0.0064 | |  | | 23 | 7 | 0.731 | 0.0065 |
| 4 | Askvoll | Norway | 2011 | 24 | | 5 | 0.493 | 0.0036 | |  | | 23 | 4 | 0.320 | 0.0025 |
| 5 | Lista | Norway | 2007 | 18 | | 10 | 0.876 | 0.0083 | |  | | 18 | 4 | 0.699 | 0.0098 |
| 6 | Jomfruland | Norway | 2004 | 13 | | 9 | 0.936 | 0,0097 | |  | | 14 | 8 | 0.769 | 0.0055 |
| 7 | Ostøya | Norway | 2006 | 22 | | 11 | 0.857 | 0.0074 | |  | | 21 | 5 | 0.490 | 0.0045 |
| 8 | Kirkøya | Norway | 2006 | 27 | | 10 | 0.858 | 0.0067 | |  | | 38 | 7 | 0.576 | 0.0081 |
| 9 | Ljungskile | Sweden | 2013 | 23 | | 12 | 0.909 | 0.0077 | |  | | 20 | 5 | 0.732 | 0.0101 |
| 10 | Falkenberg | Sweden | 2013 | 20 | | 12 | 0.905 | 0.0076 | |  | | 22 | 5 | 0.407 | 0.0052 |
| 11 | Ålbæk | Denmark | 2013 | 21 | | 11 | 0.929 | 0.0089 | |  | | 17 | 7 | 0.765 | 0.0016 |
| 12 | Fredericia | Denmark | 2013 | 20 | | 10 | 0.911 | 0.0067 | |  | | 18 | 7 | 0.745 | 0.0087 |
| 13 | Wahrzow | Germany | 2005 | 12 | | 9 | 0.939 | 0.0098 |  | | | 16 | 6 | 0.742 | 0.0093 |
| 14 | Loket | Czech Rep. | 2005 | 11 | | 8 | 0.945 | 0.0081 |  | | | 11 | 4 | 0.691 | 0.0071 |
| 15 | Gdansk | Poland | 2005 | 10 | | 7 | 0.911 | 0.0049 |  | | | 12 | 7 | 0.773 | 0.0077 |
| 16 | Helsinki | Finland | 2006 | 19 | | 6 | 0.696 | 0.0032 |  | | | 22 | 2 | 0.091 | 0.0048 |
| 17 | Mar Lodge | Scotland | 2007 | 22 | | 4 | 0.463 | 0.0035 |  | | | 22 | 5 | 0.468 | 0.0082 |
| 18 | Ballogie | Scotland | 2009 | 23 | | 8 | 0.526 | 0.0031 |  | | | 22 | 8 | 0.771 | 0.0068 |
| 19 | Glensaugh | Scotland | 2012 | 26 | | 10 | 0.671 | 0.0048 |  | | | 25 | 5 | 0.409 | 0.0059 |
| 20 | Setmurthy | England | 2013 | 19 | | 11 | 0.789 | 0.0056 |  | | | 20 | 4 | 0.489 | 0.0066 |
| 21 | Danby | England | 2013 | 20 | | 8 | 0.589 | 0.0028 |  | | | 21 | 4 | 0.271 | 0.0033 |
| 22 | Yeashley | England | 2013 | 20 | | 9 | 0.795 | 0.0067 |  | | | 16 | 4 | 0.617 | 0.0094 |
|  | Total |  |  | 430 | | 81 | 0.880 | 0.0086 |  | | | 442 | 38 | 0.698 | 0.0094 |
