# Supplementary material for: Phylogenetic Lineages and Postglacial Dispersal Dynamics Characterize the Genetic Structure of the Tick, Ixodes ricinus, in Northwest Europe
Source: PLoS One. 2016 Dec 1;11(12):e0167450. doi: 10.1371/journal.pone.0167450 (PMC5131986; doi:10.1371/journal.pone.0167450)
Supplement: S2 Table — (DOCX) [file pone.0167450.s005.docx]

Tables S2. Sampling locations, sequence name and GeneBank accession number for control region (CR) and cytochrome *b* (cyt *b)* gene of mtDNA in *Ixodus ricinus*.

| Location | Country | Location number | Sequence name | CR GenBank accession mumber | Cyt *b* GenBank accession mumber |
| --- | --- | --- | --- | --- | --- |
| Hitra | Norway | 1 | HIT348 | KY025862 | KY067031 |
| Hitra | Norway | 1 | HIT349 |  | KY067032 |
| Hitra | Norway | 1 | HIT350 | KY025863 |  |
| Hitra | Norway | 1 | HIT351 | KY025864 | KY067033 |
| Hitra | Norway | 1 | HIT352 | KY025865 |  |
| Hitra | Norway | 1 | HIT353 | KY025866 | KY067034 |
| Hitra | Norway | 1 | HIT354 | KY025867 | KY067035 |
| Hitra | Norway | 1 | HIT355 |  | KY067036 |
| Hitra | Norway | 1 | HIT356 | KY025868 | KY067037 |
| Hitra | Norway | 1 | HIT357 | KY025869 |  |
| Hitra | Norway | 1 | HIT359 | KY025870 | KY067038 |
| Hitra | Norway | 1 | HIT360 | KY025871 | KY067039 |
| Hitra | Norway | 1 | HIT361 | KY025872 | KY067040 |
| Hitra | Norway | 1 | HIT362 | KY025873 | KY067041 |
| Hitra | Norway | 1 | HIT363 | KY025874 | KY067042 |
| Hitra | Norway | 1 | HIT364 | KY025875 | KY067043 |
| Hitra | Norway | 1 | HIT365 | KY025876 | KY067044 |
| Hitra | Norway | 1 | HIT366 | KY025877 | KY067045 |
| Hitra | Norway | 1 | HIT367 | KY025878 | KY067046 |
| Hitra | Norway | 1 | HIT368 | KY025879 | KY067047 |
| Hitra | Norway | 1 | HIT369 | KY025880 | KY067048 |
| Hitra | Norway | 1 | HIT370 | KY025881 | KY067049 |
| Hareide | Norway | 2 | HAR324 |  | KY067010 |
| Hareide | Norway | 2 | HAR325 | KY025843 | KY067011 |
| Hareide | Norway | 2 | HAR326 | KY025844 | KY067012 |
| Hareide | Norway | 2 | HAR327 | KY025845 | KY067013 |
| Hareide | Norway | 2 | HAR328 |  | KY067014 |
| Hareide | Norway | 2 | HAR329 | KY025846 | KY067015 |
| Hareide | Norway | 2 | HAR331 |  | KY067016 |
| Hareide | Norway | 2 | HAR332 | KY025847 | KY067017 |
| Hareide | Norway | 2 | HAR333 | KY025848 | KY067018 |
| Hareide | Norway | 2 | HAR334 | KY025849 | KY067019 |
| Hareide | Norway | 2 | HAR335 | KY025850 | KY067020 |
| Hareide | Norway | 2 | HAR336 | KY025851 | KY067021 |
| Hareide | Norway | 2 | HAR337 | KY025852 | KY067022 |
| Hareide | Norway | 2 | HAR338 | KY025853 | KY067023 |
| Hareide | Norway | 2 | HAR339 | KY025854 | KY067024 |
| Hareide | Norway | 2 | HAR340 | KY025855 | KY067025 |
| Hareide | Norway | 2 | HAR341 | KY025856 | KY067026 |
| Hareide | Norway | 2 | HAR342 | KY025857 | KY067027 |
| Hareide | Norway | 2 | HAR343 | KY025858 | KY067028 |
| Hareide | Norway | 2 | HAR344 | KY025859 |  |
| Hareide | Norway | 2 | HAR345 | KY025860 |  |
| Hareide | Norway | 2 | HAR346 |  | KY067029 |
| Hareide | Norway | 2 | HAR347 | KY025861 | KY067030 |
| Gaular | Norway | 3 | GAU300 | KY025822 | KY066987 |
| Gaular | Norway | 3 | GAU301 | KY025823 | KY066988 |
| Gaular | Norway | 3 | GAU302 | KY025824 | KY066989 |
| Gaular | Norway | 3 | GAU303 | KY025825 | KY066990 |
| Gaular | Norway | 3 | GAU304 | KY025826 | KY066991 |
| Gaular | Norway | 3 | GAU305 | KY025827 | KY066992 |
| Gaular | Norway | 3 | GAU306 | KY025828 | KY066993 |
| Gaular | Norway | 3 | GAU307 | KY025829 | KY066994 |
| Gaular | Norway | 3 | GAU308 | KY025830 | KY066995 |
| Gaular | Norway | 3 | GAU309 | KY025831 | KY066996 |
| Gaular | Norway | 3 | GAU310 | KY025832 | KY066997 |
| Gaular | Norway | 3 | GAU311 | KY025833 | KY066998 |
| Gaular | Norway | 3 | GAU312 | KY025834 | KY066999 |
| Gaular | Norway | 3 | GAU313 | KY025835 | KY067000 |
| Gaular | Norway | 3 | GAU314 | KY025836 | KY067001 |
| Gaular | Norway | 3 | GAU315 | KY025837 | KY067002 |
| Gaular | Norway | 3 | GAU316 | KY025838 | KY067003 |
| Gaular | Norway | 3 | GAU317 | KY025839 | KY067004 |
| Gaular | Norway | 3 | GAU318 |  | KY067005 |
| Gaular | Norway | 3 | GAU319 | KY025840 | KY067006 |
| Gaular | Norway | 3 | GAU320 |  | KY067007 |
| Gaular | Norway | 3 | GAU321 | KY025841 | KY067008 |
| Gaular | Norway | 3 | GAU322 | KY025842 | KY067009 |
| Askvoll | Norway | 4 | Ask101 | KY025630 | KY066777 |
| Askvoll | Norway | 4 | Ask102 | KY025631 | KY066778 |
| Askvoll | Norway | 4 | Ask103 | KY025632 | KY066779 |
| Askvoll | Norway | 4 | Ask104 | KY025633 | KY066780 |
| Askvoll | Norway | 4 | Ask105 | KY025634 | KY066781 |
| Askvoll | Norway | 4 | Ask106 | KY025635 | KY066782 |
| Askvoll | Norway | 4 | Ask107 | KY025636 | KY066783 |
| Askvoll | Norway | 4 | Ask108 | KY025637 | KY066784 |
| Askvoll | Norway | 4 | Ask109 | KY025638 | KY066785 |
| Askvoll | Norway | 4 | Ask110 | KY025639 | KY066786 |
| Askvoll | Norway | 4 | ASK111 | KY025640 |  |
| Askvoll | Norway | 4 | Ask112 | KY025641 | KY066787 |
| Askvoll | Norway | 4 | ASK114 | KY025642 | KY066788 |
| Askvoll | Norway | 4 | Ask115 | KY025643 | KY066789 |
| Askvoll | Norway | 4 | Ask116 | KY025644 | KY066790 |
| Askvoll | Norway | 4 | Ask117 | KY025646 | KY066791 |
| Askvoll | Norway | 4 | Ask118 | KY025647 | KY066792 |
| Askvoll | Norway | 4 | Ask119 | KY025648 | KY066793 |
| Askvoll | Norway | 4 | Ask120 | KY025645 | KY066794 |
| Askvoll | Norway | 4 | Ask121 | KY025649 | KY066795 |
| Askvoll | Norway | 4 | Ask122 | KY025650 | KY066796 |
| Askvoll | Norway | 4 | Ask123 | KY025651 | KY066797 |
| Askvoll | Norway | 4 | Ask124 | KY025652 | KY066798 |
| Askvoll | Norway | 4 | Ask125 | KY025653 | KY066799 |
| Lista | Norway | 5 | LIS182 |  | KY066887 |
| Lista | Norway | 5 | LIS183 | KY025732 | KY066888 |
| Lista | Norway | 5 | LIS185 | KY025733 | KY066889 |
| Lista | Norway | 5 | LIS186 | KY025734 |  |
| Lista | Norway | 5 | LIS187 | KY025735 | KY066890 |
| Lista | Norway | 5 | LIS188 | KY025736 | KY066891 |
| Lista | Norway | 5 | LIS189 | KY025737 |  |
| Lista | Norway | 5 | LIS190 | KY025738 | KY066892 |
| Lista | Norway | 5 | LIS191 | KY025739 | KY066893 |
| Lista | Norway | 5 | LIS192 | KY025740 | KY066894 |
| Lista | Norway | 5 | LIS193 | KY025741 | KY066895 |
| Lista | Norway | 5 | LIS194 | KY025742 | KY066896 |
| Lista | Norway | 5 | LIS195 | KY025744 | KY066897 |
| Lista | Norway | 5 | LIS196 | KY025745 | KY066898 |
| Lista | Norway | 5 | LIS197 | KY025743 | KY066899 |
| Lista | Norway | 5 | LIS198 | KY025746 | KY066900 |
| Lista | Norway | 5 | LIS199 | KY025747 | KY066901 |
| Lista | Norway | 5 | LIS200 | KY025749 | KY066902 |
| Lista | Norway | 5 | LIS201 | KY025748 | KY066903 |
| Lista | Norway | 5 | LIS202 |  | KY066904 |
| Jomfruland | Norway | 6 | JOM202 | KY025595 | KY066744 |
| Jomfruland | Norway | 6 | JOM203 | KY025597 | KY066746 |
| Jomfruland | Norway | 6 | JON201 |  | KY066742 |
| Jomfruland | Norway | 6 | JON202 | KY025596 | KY066743 |
| Jomfruland | Norway | 6 | JON203 | KY025598 | KY066745 |
| Jomfruland | Norway | 6 | JON204 | KY025599 | KY066747 |
| Jomfruland | Norway | 6 | JON205 | KY025600 | KY066748 |
| Jomfruland | Norway | 6 | JON207 | KY025601 | KY066749 |
| Jomfruland | Norway | 6 | JON208 |  | KY066750 |
| Jomfruland | Norway | 6 | JON209 | KY025602 | KY066751 |
| Jomfruland | Norway | 6 | JON210 | KY025603 | KY066752 |
| Jomfruland | Norway | 6 | JON211 | KY025604 | KY066753 |
| Jomfruland | Norway | 6 | JON212 | KY025605 |  |
| Jomfruland | Norway | 6 | JON213 | KY025606 | KY066754 |
| Jomfruland | Norway | 6 | JON214 | KY025607 | KY066755 |
| Ostøya | Norway | 7 | OS_OF1 | KY025608 |  |
| Ostøya | Norway | 7 | OS_OF2 | KY025609 | KY066756 |
| Ostøya | Norway | 7 | OS_OF3 | KY025610 | KY066757 |
| Ostøya | Norway | 7 | OS_OF4 | KY025611 | KY066758 |
| Ostøya | Norway | 7 | OS_OF5 | KY025612 | KY066759 |
| Ostøya | Norway | 7 | OS_OF6 | KY025613 | KY066760 |
| Ostøya | Norway | 7 | OS_OF7 | KY025614 | KY066761 |
| Ostøya | Norway | 7 | OS_OF8 | KY025615 | KY066762 |
| Ostøya | Norway | 7 | OS_OF9 | KY025616 | KY066763 |
| Ostøya | Norway | 7 | OS_OM1 | KY025620 | KY066767 |
| Ostøya | Norway | 7 | OS_OM2 | KY025621 | KY066768 |
| Ostøya | Norway | 7 | OS_OM3 | KY025622 | KY066769 |
| Ostøya | Norway | 7 | OS_OM4 |  | KY066770 |
| Ostøya | Norway | 7 | OS_OM5 | KY025623 | KY066771 |
| Ostøya | Norway | 7 | OS_OM7 | KY025624 | KY066772 |
| Ostøya | Norway | 7 | OS_OM8 | KY025625 |  |
| Ostøya | Norway | 7 | OS_OM9 | KY025626 | KY066773 |
| Ostøya | Norway | 7 | OSOF10 | KY025617 | KY066764 |
| Ostøya | Norway | 7 | OSOF11 | KY025618 | KY066765 |
| Ostøya | Norway | 7 | OSOF12 | KY025619 | KY066766 |
| Ostøya | Norway | 7 | OSOM10 | KY025627 | KY066774 |
| Ostøya | Norway | 7 | OSOM11 | KY025628 | KY066775 |
| Ostøya | Norway | 7 | OSOM12 | KY025629 | KY066776 |
| Kirkøya | Norway | 8 | STO203 | KY025750 | KY066905 |
| Kirkøya | Norway | 8 | STO204 | KY025751 | KY066906 |
| Kirkøya | Norway | 8 | STO205 | KY025752 | KY066907 |
| Kirkøya | Norway | 8 | STO206 |  | KY066908 |
| Kirkøya | Norway | 8 | STO207 |  | KY066909 |
| Kirkøya | Norway | 8 | STO208 | KY025753 | KY066910 |
| Kirkøya | Norway | 8 | STO209 | KY025754 | KY066911 |
| Kirkøya | Norway | 8 | STO210 | KY025755 | KY066912 |
| Kirkøya | Norway | 8 | STO211 |  | KY066913 |
| Kirkøya | Norway | 8 | STO212 | KY025756 | KY066914 |
| Kirkøya | Norway | 8 | STO213 | KY025757 | KY066915 |
| Kirkøya | Norway | 8 | STO214 | KY025758 | KY066916 |
| Kirkøya | Norway | 8 | STO215 |  | KY066917 |
| Kirkøya | Norway | 8 | STO216 | KY025759 | KY066918 |
| Kirkøya | Norway | 8 | STO217 | KY025761 | KY066919 |
| Kirkøya | Norway | 8 | STO218 |  | KY066920 |
| Kirkøya | Norway | 8 | STO219 | KY025760 | KY066921 |
| Kirkøya | Norway | 8 | STO220 | KY025762 | KY066922 |
| Kirkøya | Norway | 8 | STO221 |  | KY066923 |
| Kirkøya | Norway | 8 | STO222 | KY025763 | KY066924 |
| Kirkøya | Norway | 8 | STO223 |  | KY066925 |
| Kirkøya | Norway | 8 | STO224 |  | KY066926 |
| Kirkøya | Norway | 8 | Sto225 |  | KY066927 |
| Kirkøya | Norway | 8 | STO226 |  | KY066928 |
| Kirkøya | Norway | 8 | STO227 | KY025764 | KY066929 |
| Kirkøya | Norway | 8 | STO228 | KY025765 | KY066930 |
| Kirkøya | Norway | 8 | STO229 | KY025766 | KY066931 |
| Kirkøya | Norway | 8 | STO230 | KY025767 | KY066932 |
| Kirkøya | Norway | 8 | STO231 | KY025768 |  |
| Kirkøya | Norway | 8 | STO233 |  | KY066933 |
| Kirkøya | Norway | 8 | STO234 |  | KY066934 |
| Kirkøya | Norway | 8 | STO235 | KY025769 | KY066935 |
| Kirkøya | Norway | 8 | STO236 | KY025770 | KY066936 |
| Kirkøya | Norway | 8 | STO237 | KY025771 |  |
| Kirkøya | Norway | 8 | STO239 | KY025772 | KY066937 |
| Kirkøya | Norway | 8 | STO240 |  | KY066938 |
| Kirkøya | Norway | 8 | STO241 | KY025773 | KY066939 |
| Kirkøya | Norway | 8 | STO243 | KY025774 | KY066940 |
| Kirkøya | Norway | 8 | STO244 | KY025775 | KY066941 |
| Kirkøya | Norway | 8 | STO245 | KY025776 | KY066942 |
| Ljungskila | Sweden | 9 | SNG516 | KY026002 | KY067164 |
| Ljungskila | Sweden | 9 | SNG517 | KY026003 | KY067165 |
| Ljungskila | Sweden | 9 | SNG518 | KY026004 | KY067166 |
| Ljungskila | Sweden | 9 | SNG519 | KY026005 | KY067167 |
| Ljungskila | Sweden | 9 | SNG520 | KY026006 | KY067168 |
| Ljungskila | Sweden | 9 | SNG521 | KY026007 |  |
| Ljungskila | Sweden | 9 | SNG522 | KY026008 | KY067169 |
| Ljungskila | Sweden | 9 | SNG523 | KY026009 | KY067170 |
| Ljungskila | Sweden | 9 | SNG524 | KY026010 | KY067171 |
| Ljungskila | Sweden | 9 | SNG525 | KY026011 | KY067172 |
| Ljungskila | Sweden | 9 | SNG526 | KY026012 | KY067173 |
| Ljungskila | Sweden | 9 | SNG527 | KY026013 | KY067174 |
| Ljungskila | Sweden | 9 | SNG528 | KY026014 | KY067175 |
| Ljungskila | Sweden | 9 | SNG529 |  | KY067176 |
| Ljungskila | Sweden | 9 | SNG530 | KY026015 |  |
| Ljungskila | Sweden | 9 | SNG531 | KY026016 | KY067177 |
| Ljungskila | Sweden | 9 | SNG532 | KY026017 | KY067178 |
| Ljungskila | Sweden | 9 | SNG533 | KY026018 | KY067179 |
| Ljungskila | Sweden | 9 | SNG534 | KY026019 | KY067180 |
| Ljungskila | Sweden | 9 | SNG535 | KY026020 | KY067181 |
| Ljungskila | Sweden | 9 | SNG536 | KY026021 | KY067182 |
| Ljungskila | Sweden | 9 | SNG537 | KY026022 | KY067183 |
| Ljungskila | Sweden | 9 | SNG538 | KY026023 |  |
| Ljungskila | Sweden | 9 | SNG539 | KY026024 |  |
| Falkenberg | Sweden | 10 | SSG492 |  | KY067142 |
| Falkenberg | Sweden | 10 | SSG493 | KY025982 | KY067143 |
| Falkenberg | Sweden | 10 | SSG494 | KY025983 | KY067144 |
| Falkenberg | Sweden | 10 | SSG495 | KY025984 | KY067145 |
| Falkenberg | Sweden | 10 | SSG496 | KY025985 |  |
| Falkenberg | Sweden | 10 | SSG497 | KY025986 | KY067146 |
| Falkenberg | Sweden | 10 | SSG498 | KY025987 | KY067147 |
| Falkenberg | Sweden | 10 | SSG499 | KY025988 | KY067148 |
| Falkenberg | Sweden | 10 | SSG500 | KY025989 | KY067149 |
| Falkenberg | Sweden | 10 | SSG501 | KY025990 | KY067150 |
| Falkenberg | Sweden | 10 | SSG502 | KY025991 | KY067151 |
| Falkenberg | Sweden | 10 | SSG503 | KY025992 | KY067152 |
| Falkenberg | Sweden | 10 | SSG504 | KY025993 | KY067153 |
| Falkenberg | Sweden | 10 | SSG505 |  | KY067154 |
| Falkenberg | Sweden | 10 | SSG506 | KY025994 | KY067155 |
| Falkenberg | Sweden | 10 | SSG507 | KY025995 | KY067156 |
| Falkenberg | Sweden | 10 | SSG508 | KY025996 | KY067157 |
| Falkenberg | Sweden | 10 | SSG509 | KY025997 | KY067158 |
| Falkenberg | Sweden | 10 | SSG510 | KY025998 | KY067159 |
| Falkenberg | Sweden | 10 | SSG511 |  | KY067160 |
| Falkenberg | Sweden | 10 | SSG512 | KY025999 | KY067161 |
| Falkenberg | Sweden | 10 | SSG513 | KY026000 | KY067162 |
| Falkenberg | Sweden | 10 | SSG514 | KY026001 | KY067163 |
| Ålbæk | Denmark | 11 | DNJ445 | KY025941 |  |
| Ålbæk | Denmark | 11 | DNJ446 | KY025942 |  |
| Ålbæk | Denmark | 11 | DNJ447 | KY025943 |  |
| Ålbæk | Denmark | 11 | DNJ448 | KY025944 | KY067107 |
| Ålbæk | Denmark | 11 | DNJ449 | KY025945 | KY067108 |
| Ålbæk | Denmark | 11 | DNJ450 | KY025946 | KY067109 |
| Ålbæk | Denmark | 11 | DNJ451 |  | KY067110 |
| Ålbæk | Denmark | 11 | DNJ452 | KY025947 | KY067111 |
| Ålbæk | Denmark | 11 | DNJ453 | KY025948 | KY067112 |
| Ålbæk | Denmark | 11 | DNJ454 | KY025949 | KY067113 |
| Ålbæk | Denmark | 11 | DNJ455 | KY025950 | KY067114 |
| Ålbæk | Denmark | 11 | DNJ456 | KY025951 | KY067115 |
| Ålbæk | Denmark | 11 | DNJ457 |  | KY067116 |
| Ålbæk | Denmark | 11 | DNJ458 | KY025952 | KY067117 |
| Ålbæk | Denmark | 11 | DNJ459 | KY025953 | KY067118 |
| Ålbæk | Denmark | 11 | DNJ460 | KY025954 | KY067119 |
| Ålbæk | Denmark | 11 | DNJ461 | KY025955 | KY067120 |
| Ålbæk | Denmark | 11 | DNJ462 | KY025956 | KY067121 |
| Ålbæk | Denmark | 11 | DNJ463 | KY025957 | KY067122 |
| Ålbæk | Denmark | 11 | DNJ464 | KY025958 |  |
| Ålbæk | Denmark | 11 | DNJ465 | KY025959 |  |
| Ålbæk | Denmark | 11 | DNJ466 | KY025960 |  |
| Ålbæk | Denmark | 11 | DNJ467 | KY025961 |  |
| Ålbæk | Denmark | 11 | DNJ468 |  | KY067123 |
| Fredericia | Denmark | 12 | DSJ469 | KY025962 |  |
| Fredericia | Denmark | 12 | DSJ471 | KY025963 | KY067124 |
| Fredericia | Denmark | 12 | DSJ472 | KY025964 | KY067125 |
| Fredericia | Denmark | 12 | DSJ473 | KY025965 | KY067126 |
| Fredericia | Denmark | 12 | DSJ474 | KY025966 | KY067127 |
| Fredericia | Denmark | 12 | DSJ475 | KY025967 | KY067128 |
| Fredericia | Denmark | 12 | DSJ476 | KY025968 | KY067129 |
| Fredericia | Denmark | 12 | DSJ477 | KY025969 | KY067130 |
| Fredericia | Denmark | 12 | DSJ478 | KY025970 | KY067131 |
| Fredericia | Denmark | 12 | DSJ479 | KY025971 | KY067132 |
| Fredericia | Denmark | 12 | DSJ480 | KY025972 | KY067133 |
| Fredericia | Denmark | 12 | DSJ481 | KY025973 | KY067134 |
| Fredericia | Denmark | 12 | DSJ482 | KY025974 | KY067135 |
| Fredericia | Denmark | 12 | DSJ483 |  | KY067136 |
| Fredericia | Denmark | 12 | DSJ484 | KY025975 | KY067137 |
| Fredericia | Denmark | 12 | DSJ485 | KY025976 | KY067138 |
| Fredericia | Denmark | 12 | DSJ486 | KY025977 | KY067139 |
| Fredericia | Denmark | 12 | DSJ488 | KY025978 |  |
| Fredericia | Denmark | 12 | DSJ489 | KY025979 |  |
| Fredericia | Denmark | 12 | DSJ490 |  | KY067140 |
| Fredericia | Denmark | 12 | DSJ491 | KY025980 | KY067141 |
| Fredericia | Denmark | 12 | DSJ492 | KY025981 |  |
| Wahrzow | Germany | 13 | TYS_45 |  | KY066826 |
| Wahrzow | Germany | 13 | TYS_46 |  | KY066827 |
| Wahrzow | Germany | 13 | TYS_47 |  | KY066828 |
| Wahrzow | Germany | 13 | TYS_49 | KY025681 |  |
| Wahrzow | Germany | 13 | TYS_50 | KY025682 | KY066829 |
| Wahrzow | Germany | 13 | TYS_51 | KY025680 | KY066830 |
| Wahrzow | Germany | 13 | TYS_52 | KY025683 | KY066831 |
| Wahrzow | Germany | 13 | TYS_53 | KY025689 | KY066832 |
| Wahrzow | Germany | 13 | TYS_54 | KY025684 | KY066833 |
| Wahrzow | Germany | 13 | TYS_55 | KY025686 | KY066834 |
| Wahrzow | Germany | 13 | TYS_56 | KY025688 |  |
| Wahrzow | Germany | 13 | TYS_57 | KY025685 | KY066835 |
| Wahrzow | Germany | 13 | TYS_58 |  | KY066836 |
| Wahrzow | Germany | 13 | TYS_59 |  | KY066837 |
| Wahrzow | Germany | 13 | TYS_60 | KY025687 | KY066838 |
| Wahrzow | Germany | 13 | TYS_61 | KY025690 | KY066839 |
| Wahrzow | Germany | 13 | TYS_63 | KY025691 | KY066840 |
| Wahrzow | Germany | 13 | TYS_64 |  | KY066841 |
| Loket | Czech | 14 | CHE123 | KY025692 | KY066842 |
| Loket | Czech | 14 | CHE124 | KY025694 | KY066843 |
| Loket | Czech | 14 | CHE125 | KY025693 | KY066844 |
| Loket | Czech | 14 | CHE126 |  | KY066845 |
| Loket | Czech | 14 | CHE127 |  | KY066846 |
| Loket | Czech | 14 | CHE129 | KY025695 | KY066847 |
| Loket | Czech | 14 | CHE130 | KY025696 |  |
| Loket | Czech | 14 | CHE131 | KY025697 | KY066848 |
| Loket | Czech | 14 | CHE132 | KY025698 | KY066849 |
| Loket | Czech | 14 | CHE133 | KY025699 | KY066850 |
| Loket | Czech | 14 | CHE134 | KY025700 | KY066851 |
| Loket | Czech | 14 | CHE137 | KY025701 | KY066852 |
| Loket | Czech | 14 | CHE138 | KY025702 |  |
| Gdansk | Poland | 15 | POL101 |  | KY066853 |
| Gdansk | Poland | 15 | POL102 |  | KY066854 |
| Gdansk | Poland | 15 | POL103 | KY025704 | KY066855 |
| Gdansk | Poland | 15 | POL104 | KY025705 | KY066856 |
| Gdansk | Poland | 15 | POL105 | KY025710 |  |
| Gdansk | Poland | 15 | POL106 | KY025711 |  |
| Gdansk | Poland | 15 | POL108 |  | KY066857 |
| Gdansk | Poland | 15 | POL109 |  | KY066858 |
| Gdansk | Poland | 15 | POL111 | KY025706 | KY066859 |
| Gdansk | Poland | 15 | POL112 | KY025707 | KY066860 |
| Gdansk | Poland | 15 | POL113 |  | KY066861 |
| Gdansk | Poland | 15 | POL114 |  | KY066862 |
| Gdansk | Poland | 15 | POL115 |  | KY066863 |
| Gdansk | Poland | 15 | POL117 | KY025708 |  |
| Gdansk | Poland | 15 | POL118 | KY025709 |  |
| Gdansk | Poland | 15 | POL119 |  | KY066864 |
| Gdansk | Poland | 15 | POL121 | KY025703 |  |
| Gdansk | Poland | 15 | POL122 | KY025712 |  |
| Helsinki | Finland | 16 | FIN126 | KY025713 | KY066865 |
| Helsinki | Finland | 16 | FIN127 | KY025714 | KY066866 |
| Helsinki | Finland | 16 | FIN128 | KY025715 | KY066867 |
| Helsinki | Finland | 16 | FIN129 | KY025716 | KY066868 |
| Helsinki | Finland | 16 | FIN130 | KY025717 | KY066869 |
| Helsinki | Finland | 16 | FIN131 | KY025718 | KY066870 |
| Helsinki | Finland | 16 | FIN132 | KY025719 | KY066871 |
| Helsinki | Finland | 16 | FIN133 | KY025720 |  |
| Helsinki | Finland | 16 | FIN134 | KY025721 | KY066872 |
| Helsinki | Finland | 16 | FIN135 | KY025722 | KY066873 |
| Helsinki | Finland | 16 | FIN136 |  | KY066874 |
| Helsinki | Finland | 16 | FIN137 | KY025723 | KY066875 |
| Helsinki | Finland | 16 | FIN138 | KY025724 | KY066876 |
| Helsinki | Finland | 16 | FIN139 | KY025725 | KY066877 |
| Helsinki | Finland | 16 | FIN140 | KY025726 | KY066878 |
| Helsinki | Finland | 16 | FIN141 | KY025727 | KY066879 |
| Helsinki | Finland | 16 | FIN143 |  | KY066880 |
| Helsinki | Finland | 16 | FIN144 | KY025728 | KY066881 |
| Helsinki | Finland | 16 | FIN145 | KY025730 | KY066882 |
| Helsinki | Finland | 16 | FIN146 | KY025729 | KY066883 |
| Helsinki | Finland | 16 | FIN147 | KY025731 | KY066884 |
| Helsinki | Finland | 16 | FIN148 |  | KY066885 |
| Helsinki | Finland | 16 | FIN149 |  | KY066886 |
| Mar Lodge | Scotland | 17 | SKC275 |  | KY066943 |
| Mar Lodge | Scotland | 17 | SKC276 |  | KY066944 |
| Mar Lodge | Scotland | 17 | SKC277 | KY025777 | KY066945 |
| Mar Lodge | Scotland | 17 | SKC278 | KY025778 | KY066946 |
| Mar Lodge | Scotland | 17 | SKC279 | KY025779 | KY066947 |
| Mar Lodge | Scotland | 17 | SKC280 | KY025780 | KY066948 |
| Mar Lodge | Scotland | 17 | SKC281 | KY025781 | KY066949 |
| Mar Lodge | Scotland | 17 | SKC282 | KY025782 | KY066950 |
| Mar Lodge | Scotland | 17 | SKC283 |  | KY066951 |
| Mar Lodge | Scotland | 17 | SKC284 | KY025783 | KY066952 |
| Mar Lodge | Scotland | 17 | SKC285 | KY025784 | KY066953 |
| Mar Lodge | Scotland | 17 | SKC286 | KY025785 | KY066954 |
| Mar Lodge | Scotland | 17 | SKC287 | KY025786 | KY066955 |
| Mar Lodge | Scotland | 17 | SKC288 | KY025787 | KY066956 |
| Mar Lodge | Scotland | 17 | SKC289 | KY025788 | KY066957 |
| Mar Lodge | Scotland | 17 | SKC290 | KY025789 | KY066958 |
| Mar Lodge | Scotland | 17 | SKC291 | KY025790 |  |
| Mar Lodge | Scotland | 17 | SKC292 | KY025791 | KY066959 |
| Mar Lodge | Scotland | 17 | SKC293 | KY025792 | KY066960 |
| Mar Lodge | Scotland | 17 | SKC294 | KY025793 | KY066961 |
| Mar Lodge | Scotland | 17 | SKC295 | KY025794 |  |
| Mar Lodge | Scotland | 17 | SKC296 | KY025795 | KY066962 |
| Mar Lodge | Scotland | 17 | SKC297 | KY025797 | KY066963 |
| Mar Lodge | Scotland | 17 | SKC298 | KY025796 | KY066964 |
| Mar Lodge | Scotland | 17 | SKC299 | KY025798 |  |
| Ballogie | Scotland | 18 | SKB252 | KY025799 | KY066965 |
| Ballogie | Scotland | 18 | SKB253 | KY025800 | KY066966 |
| Ballogie | Scotland | 18 | SKB254 | KY025801 | KY066967 |
| Ballogie | Scotland | 18 | SKB255 | KY025802 | KY066968 |
| Ballogie | Scotland | 18 | SKB256 | KY025803 | KY066969 |
| Ballogie | Scotland | 18 | SKB257 | KY025804 | KY066970 |
| Ballogie | Scotland | 18 | SKB258 | KY025805 | KY066971 |
| Ballogie | Scotland | 18 | SKB259 | KY025806 | KY066972 |
| Ballogie | Scotland | 18 | SKB260 | KY025807 | KY066973 |
| Ballogie | Scotland | 18 | SKB261 | KY025808 | KY066974 |
| Ballogie | Scotland | 18 | SKB263 | KY025809 | KY066975 |
| Ballogie | Scotland | 18 | SKB264 | KY025810 | KY066976 |
| Ballogie | Scotland | 18 | SKB265 | KY025811 | KY066977 |
| Ballogie | Scotland | 18 | SKB266 | KY025812 | KY066978 |
| Ballogie | Scotland | 18 | SKB267 | KY025813 | KY066979 |
| Ballogie | Scotland | 18 | SKB268 | KY025814 | KY066980 |
| Ballogie | Scotland | 18 | SKB269 | KY025815 | KY066981 |
| Ballogie | Scotland | 18 | SKB270 | KY025816 | KY066982 |
| Ballogie | Scotland | 18 | SKB271 | KY025817 | KY066983 |
| Ballogie | Scotland | 18 | SKB272 | KY025818 | KY066984 |
| Ballogie | Scotland | 18 | SKB273 | KY025819 | KY066985 |
| Ballogie | Scotland | 18 | SKB274 | KY025820 | KY066986 |
| Ballogie | Scotland | 18 | SKCB75 | KY025821 |  |
| Glensaugh | Scotland | 19 | SKO150 | KY025654 | KY066800 |
| Glensaugh | Scotland | 19 | SKO151 | KY025655 | KY066801 |
| Glensaugh | Scotland | 19 | SKO152 | KY025656 | KY066802 |
| Glensaugh | Scotland | 19 | SKO153 | KY025657 | KY066803 |
| Glensaugh | Scotland | 19 | SKO155 | KY025658 | KY066804 |
| Glensaugh | Scotland | 19 | SKO157 | KY025659 | KY066805 |
| Glensaugh | Scotland | 19 | SKO158 | KY025660 | KY066806 |
| Glensaugh | Scotland | 19 | sko159 | KY025661 | KY066807 |
| Glensaugh | Scotland | 19 | SKO160 | KY025662 | KY066808 |
| Glensaugh | Scotland | 19 | SKO161 | KY025663 | KY066809 |
| Glensaugh | Scotland | 19 | SKO163 | KY025664 | KY066810 |
| Glensaugh | Scotland | 19 | SKO164 | KY025665 | KY066811 |
| Glensaugh | Scotland | 19 | SKO165 | KY025666 | KY066812 |
| Glensaugh | Scotland | 19 | SKO166 | KY025667 | KY066813 |
| Glensaugh | Scotland | 19 | SKO167 | KY025668 | KY066814 |
| Glensaugh | Scotland | 19 | SKO168 | KY025669 | KY066815 |
| Glensaugh | Scotland | 19 | SKO169 | KY025670 | KY066816 |
| Glensaugh | Scotland | 19 | SKO170 | KY025671 |  |
| Glensaugh | Scotland | 19 | SKO171 |  | KY066817 |
| Glensaugh | Scotland | 19 | SKO172 | KY025672 | KY066818 |
| Glensaugh | Scotland | 19 | SKO173 | KY025673 | KY066819 |
| Glensaugh | Scotland | 19 | SKO174 | KY025674 | KY066820 |
| Glensaugh | Scotland | 19 | SKO176 | KY025675 | KY066821 |
| Glensaugh | Scotland | 19 | SKO177 | KY025676 | KY066822 |
| Glensaugh | Scotland | 19 | SKO179 | KY025677 | KY066823 |
| Glensaugh | Scotland | 19 | SKO180 | KY025678 | KY066824 |
| Glensaugh | Scotland | 19 | SKO181 | KY025679 | KY066825 |
| Setmurthy | England | 20 | UKI420 | KY025902 | KY067071 |
| Setmurthy | England | 20 | UKI421 | KY025903 | KY067072 |
| Setmurthy | England | 20 | UKI422 | KY025904 | KY067073 |
| Setmurthy | England | 20 | UKI423 | KY025905 | KY067074 |
| Setmurthy | England | 20 | UKI424 |  | KY067075 |
| Setmurthy | England | 20 | UKI425 | KY025906 |  |
| Setmurthy | England | 20 | UKI426 |  | KY067076 |
| Setmurthy | England | 20 | UKI428 | KY025907 |  |
| Setmurthy | England | 20 | UKI429 | KY025908 |  |
| Setmurthy | England | 20 | UKI430 | KY025909 | KY067077 |
| Setmurthy | England | 20 | UKI431 | KY025910 | KY067078 |
| Setmurthy | England | 20 | UKI432 | KY025911 | KY067079 |
| Setmurthy | England | 20 | UKI433 | KY025912 | KY067080 |
| Setmurthy | England | 20 | UKI434 | KY025913 | KY067081 |
| Setmurthy | England | 20 | UKI435 | KY025914 | KY067082 |
| Setmurthy | England | 20 | UKI436 | KY025915 | KY067083 |
| Setmurthy | England | 20 | UKI437 | KY025916 | KY067084 |
| Setmurthy | England | 20 | UKI438 |  | KY067085 |
| Setmurthy | England | 20 | UKI439 | KY025917 | KY067086 |
| Setmurthy | England | 20 | UKI440 | KY025918 | KY067087 |
| Setmurthy | England | 20 | UKI441 | KY025919 | KY067088 |
| Setmurthy | England | 20 | UKI442 | KY025920 | KY067089 |
| Setmurthy | England | 20 | UKI443 |  | KY067090 |
| Danby | England | 21 | UKD372 | KY025882 | KY067050 |
| Danby | England | 21 | UKD373 | KY025897 | KY067051 |
| Danby | England | 21 | UKD374 | KY025883 | KY067052 |
| Danby | England | 21 | UKD375 | KY025884 | KY067053 |
| Danby | England | 21 | UKD376 | KY025898 | KY067054 |
| Danby | England | 21 | UKD377 |  | KY067055 |
| Danby | England | 21 | UKD378 | KY025885 | KY067056 |
| Danby | England | 21 | UKD379 | KY025886 | KY067057 |
| Danby | England | 21 | UKD380 | KY025887 | KY067058 |
| Danby | England | 21 | UKD381 | KY025888 | KY067059 |
| Danby | England | 21 | UKD382 | KY025889 | KY067060 |
| Danby | England | 21 | UKD384 | KY025890 | KY067061 |
| Danby | England | 21 | UKD385 |  | KY067062 |
| Danby | England | 21 | UKD386 | KY025899 | KY067063 |
| Danby | England | 21 | UKD387 | KY025900 | KY067064 |
| Danby | England | 21 | UKD388 | KY025901 | KY067065 |
| Danby | England | 21 | UKD389 | KY025891 | KY067066 |
| Danby | England | 21 | UKD390 | KY025892 | KY067067 |
| Danby | England | 21 | UKD391 | KY025893 |  |
| Danby | England | 21 | UKD392 | KY025894 | KY067068 |
| Danby | England | 21 | UKD393 | KY025895 | KY067069 |
| Danby | England | 21 | UKD394 | KY025896 | KY067070 |
| Yeashley | England | 22 | UKM396 | KY025921 | KY067091 |
| Yeashley | England | 22 | UKM397 | KY025922 | KY067092 |
| Yeashley | England | 22 | UKM398 | KY025923 | KY067093 |
| Yeashley | England | 22 | UKM399 | KY025924 | KY067094 |
| Yeashley | England | 22 | UKM400 | KY025925 |  |
| Yeashley | England | 22 | UKM402 | KY025926 |  |
| Yeashley | England | 22 | UKM405 | KY025927 |  |
| Yeashley | England | 22 | UKM406 | KY025928 |  |
| Yeashley | England | 22 | UKM407 | KY025929 |  |
| Yeashley | England | 22 | UKM408 | KY025930 | KY067095 |
| Yeashley | England | 22 | UKM409 | KY025931 | KY067096 |
| Yeashley | England | 22 | UKM410 | KY025932 | KY067097 |
| Yeashley | England | 22 | UKM411 | KY025933 | KY067098 |
| Yeashley | England | 22 | UKM412 |  | KY067099 |
| Yeashley | England | 22 | UKM413 | KY025934 | KY067100 |
| Yeashley | England | 22 | UKM414 | KY025935 | KY067101 |
| Yeashley | England | 22 | UKM415 | KY025936 | KY067102 |
| Yeashley | England | 22 | UKM416 | KY025937 | KY067103 |
| Yeashley | England | 22 | UKM417 | KY025938 | KY067104 |
| Yeashley | England | 22 | UKM418 | KY025939 | KY067105 |
| Yeashley | England | 22 | UKM419 | KY025940 | KY067106 |
