# Supplementary material for: Phylogenetic Lineages and Postglacial Dispersal Dynamics Characterize the Genetic Structure of the Tick, Ixodes ricinus, in Northwest Europe
Source: PLoS One. 2016 Dec 1;11(12):e0167450. doi: 10.1371/journal.pone.0167450 (PMC5131986; doi:10.1371/journal.pone.0167450)
Supplement: S3 Table — (DOCX) [file pone.0167450.s006.docx]

S3 Table. Pairwise genetic differences in control region of mtDNA between samples of *Ixodus ricinus* from different locations in northern Europe. F_ST_-values below the diagonal with corresponding significance levels above the diagonal (ns = non-significant, *** = P <0.001, ** = 0.001<P<0.01, * = 0.01< P <0.05). Location numbers refers to locations in Fig. 1 in main manuscript.

| Location | 1 | 2 | 3 | 4 | 5 | 6 | 7 | 8 | 9 | 10 | 11 | 12 | 13 | 14 | 15 | 16 | 17 | 18 | 19 | 20 | 21 | 22 |
| --- | --- | --- | --- | --- | --- | --- | --- | --- | --- | --- | --- | --- | --- | --- | --- | --- | --- | --- | --- | --- | --- | --- |
| 01 |  | * | ns | *** | * | *** | *** | *** | *** | *** | *** | *** | *** | *** | *** | *** | *** | *** | *** | *** | *** | *** |
| 02 | .171 |  | ns | ns | ns | *** | *** | *** | *** | *** | *** | *** | *** | *** | *** | *** | *** | *** | *** | *** | *** | *** |
| 03 | .083 | .000 |  | ns | ns | *** | *** | *** | *** | *** | *** | *** | *** | *** | *** | *** | *** | *** | *** | *** | *** | *** |
| 04 | .212 | .000 | .025 |  | * | *** | *** | *** | *** | *** | *** | *** | *** | *** | *** | *** | *** | *** | *** | *** | *** | *** |
| 05 | .101 | .096 | .000 | .146 |  | * | *** | *** | *** | *** | *** | *** | *** | *** | *** | *** | *** | *** | *** | *** | *** | *** |
| 06 | .208 | .204 | .137 | .253 | .081 |  | ns | *** | * | ns | ns | ns | ns | * | ns | ** | *** | *** | *** | *** | *** | *** |
| 07 | .357 | .407 | .307 | .454 | .193 | .024 |  | ns | ns | ns | ns | ns | ns | ns | ns | ns | *** | *** | *** | *** | *** | *** |
| 08 | .448 | .479 | .371 | .523 | .236 | .146 | .032 |  | ns | * | ns | ns | ns | ns | ns | * | *** | *** | *** | *** | *** | *** |
| 09 | .382 | .417 | .302 | .467 | .159 | .093 | .012 | .000 |  | ns | ns | ns | ns | ns | ns | ** | *** | *** | *** | *** | *** | *** |
| 10 | .386 | .433 | .335 | .479 | .219 | .038 | .000 | .054 | .032 |  | ns | ns | ns | ns | ns | ns | *** | *** | *** | *** | *** | *** |
| 11 | .367 | .411 | .313 | .460 | .192 | .048 | .000 | .038 | .016 | .000 |  | ns | ns | ns | ns | * | *** | *** | *** | *** | *** | *** |
| 12 | .356 | .411 | .287 | .464 | .148 | .055 | .000 | .003 | .000 | .000 | .000 |  | ns | ns | ns | ** | *** | *** | *** | *** | *** | *** |
| 13 | .382 | .402 | .274 | .461 | .121 | .080 | .031 | .000 | .000 | .035 | .002 | .000 |  | ns | ns | *** | *** | *** | *** | * | *** | * |
| 14 | .493 | .514 | .399 | .566 | .263 | .087 | .000 | .017 | .000 | .000 | .000 | .008 | .005 |  | ns | ns | *** | *** | *** | *** | *** | *** |
| 15 | .552 | .590 | .458 | .636 | .314 | .146 | .009 | .025 | .016 | .011 | .036 | .022 | .040 | .000 |  | ns | *** | *** | *** | *** | *** | *** |
| 16 | .611 | .641 | .527 | .675 | .404 | .192 | .028 | .094 | .089 | .026 | .071 | .089 | .138 | .000 | .000 |  | *** | *** | *** | *** | *** | *** |
| 17 | .619 | .595 | .448 | .636 | .297 | .472 | .466 | .377 | .314 | .489 | .408 | .394 | .254 | .481 | .570 | .647 |  | ns | ns | ns | ns | ns |
| 18 | .649 | .641 | .498 | .678 | .345 | .507 | .485 | .389 | .328 | .507 | .424 | .412 | .275 | .499 | .588 | .663 | .000 |  | ns | ns | ns | ns |
| 19 | .563 | .553 | .415 | .596 | .266 | .435 | .428 | .336 | .275 | .441 | .370 | .344 | .213 | .429 | .500 | .585 | .000 | .000 |  | ns | ns | ns |
| 20 | .545 | .533 | .394 | .582 | .239 | .365 | .351 | .255 | .200 | .370 | .287 | .265 | .131 | .332 | .417 | .517 | .002 | .000 | .000 |  | ns | ns |
| 21 | .675 | .664 | .520 | .700 | .370 | .526 | .507 | .416 | .355 | .529 | .444 | .442 | .301 | .523 | .619 | .691 | .000 | .000 | .004 | .023 |  | ns |
| 22 | .471 | .459 | .329 | .513 | .167 | .300 | .291 | .208 | .146 | .313 | .245 | .204 | .083 | .278 | .343 | .449 | .010 | .025 | .003 | .000 | .045 |  |
