# Supplementary material for: Phylogenetic Lineages and Postglacial Dispersal Dynamics Characterize the Genetic Structure of the Tick, Ixodes ricinus, in Northwest Europe
Source: PLoS One. 2016 Dec 1;11(12):e0167450. doi: 10.1371/journal.pone.0167450 (PMC5131986; doi:10.1371/journal.pone.0167450)
Supplement: S4 Table — (DOCX) [file pone.0167450.s007.docx]

S4 Table. Pairwise genetic differences in cytochrome *b* gene of mtDNA between samples of *Ixodus ricinus* from different locations in northern Europe. F_ST_-values below the diagonal with corresponding significance levels above the diagonal (ns = non-significant, *** = P <0.001, ** = 0.001<P<0.01, * = 0.01< P <0.05). Location numbers refers to locations in Fig. 1 in main manuscript.

| Loc-ation | 1 | 2 | 3 | 4 | 5 | 6 | 7 | 8 | 9 | 10 | 11 | 12 | 13 | 14 | 15 | 16 | 17 | 18 | 19 | 20 | 21 | 22 |
| --- | --- | --- | --- | --- | --- | --- | --- | --- | --- | --- | --- | --- | --- | --- | --- | --- | --- | --- | --- | --- | --- | --- |
| 01 |  | * | ** | ** | *** | *** | *** | *** | *** | *** | *** | *** | *** | *** | *** | *** | *** | *** | *** | *** | *** | *** |
| 02 | .165 |  | ns | Ns | * | ns | ns | ns | * | ns | ns | ns | ns | ns | ns | ns | *** | *** | *** | *** | *** | *** |
| 03 | .161 | .000 |  | Ns | ns | ns | ns | ns | ns | ns | ns | ns | ns | ns | Ns | ns | *** | *** | *** | *** | *** | *** |
| 04 | .247 | .000 | .070 |  | ** | ns | ns | *** | *** | ns | * | * | *** | * | ns | ns | *** | *** | *** | *** | *** | *** |
| 05 | .329 | .132 | .084 | .249 |  | * | ns | ns | ns | * | ns | ns | ns | * | ns | ** | ** | *** | *** | *** | *** | ns |
| 06 | .184 | .000 | .012 | .000 | .137 |  | ns | ns | ns | ns | ns | ns | ns | ns | ns | ns | *** | *** | *** | *** | *** | ** |
| 07 | .153 | .000 | .000 | .000 | .130 | .000 |  | ns | ns | ns | ns | ns | ns | ns | ns | ns | *** | *** | *** | *** | *** | *** |
| 08 | .275 | .061 | .005 | .138 | .000 | .065 | .062 |  | ns | ns | ns | ns | ns | ns | ns | * | *** | *** | *** | *** | *** | * |
| 09 | .304 | .110 | .017 | .214 | .000 | .107 | .109 | .000 |  | ns | ns | ns | ns | ns | ns | *** | *** | *** | *** | *** | *** | ns |
| 10 | .239 | .000 | .000 | .033 | .071 | .000 | .000 | .000 | .054 |  | ns | ns | ns | ns | ns | ns | *** | *** | *** | *** | *** | ** |
| 11 | .287 | .042 | .000 | .141 | .000 | .046 | .041 | .010 | .000 | .000 |  | ns | ns | ns | ns | * | *** | *** | *** | *** | *** | * |
| 12 | .204 | .000 | .000 | .070 | .009 | .000 | .000 | .000 | .000 | .000 | .000 |  | ns | ns | ns | ns | *** | *** | *** | *** | *** | * |
| 13 | .338 | .094 | .000 | .203 | .000 | .084 | .093 | .000 | .000 | .032 | .000 | .000 |  | ns | ns | * | *** | *** | *** | ** | *** | Ns |
| 14 | .293 | .051 | .010 | .084 | .050 | .000 | .005 | .000 | .028 | .000 | .000 | .000 | .013 |  | ns | * | *** | *** | *** | *** | *** | * |
| 15 | .257 | .000 | .000 | .057 | .037 | .000 | .000 | .000 | .022 | .000 | .000 | .000 | .000 | .000 |  | * | *** | *** | *** | *** | *** | * |
| 16 | .357 | .000 | .085 | .000 | 257 | .000 | .000 | .130 | .216 | .029 | .142 | .075 | .200 | .079 | .050 |  | *** | *** | *** | *** | *** | *** |
| 17 | .742 | .621 | .484 | .709 | .282 | .605 | .617 | .389 | .302 | .556 | .414 | .462 | .342 | .518 | .500 | .717 |  | ns | ns | ns | ns | Ns |
| 18 | .834 | .735 | .609 | .811 | .427 | .724 | .731 | .511 | .440 | .681 | .557 | .592 | .489 | .661 | .640 | .821 | .001 |  | ns | ns | ns | * |
| 19 | .758 | .648 | .519 | .731 | .317 | .639 | .645 | .423 | .334 | .591 | .453 | .498 | .380 | .564 | .544 | .739 | .000 | .017 |  | ns | ns | ns |
| 20 | .732 | .600 | .600 | .696 | .241 | .584 | .595 | .353 | .260 | .532 | .378 | .429 | .301 | .494 | .472 | .705 | .000 | .037 | .000 |  | ns | Ns |
| 21 | .859 | .756 | .625 | .835 | .437 | .748 | .752 | .515 | .444 | .700 | .573 | .607 | .500 | .691 | .664 | .846 | .038 | .015 | .000 | .020 |  | ** |
| 22 | .579 | .403 | .248 | .525 | .045 | .385 | .398 | .156 | 066 | .324 | .160 | .218 | .093 | .275 | .255 | .533 | .053 | .185 | .071 | .011 | .180 |  |
